# A carbene-stabilized diphosphorus: a triple-bonded diphosphorus (P≡P) and a bis(phosphinidene) (P–P) transfer agent†

**DOI:** 10.1039/d4sc05091f

**Published:** 2024-09-02

**Authors:** Joseph S. Yoon, Mehdi Abdellaoui, Milan Gembicky, Guy Bertrand

**Affiliations:** a UCSD-CNRS Joint Research Laboratory (IRL 3555), Department of Chemistry and Biochemistry, University of California San Diego La Jolla CA 92093-0358 USA gbertrand@ucsd.edu

## Abstract

The reaction of the *N*,*N*-diisopropyl bromoiminium salt with excess sodium phosphaethynolate (NaPCO) affords a diphospha-urea 2. Under blue light irradiation (450 nm), carbon monoxide is liberated affording the bis(carbene)P_2_ adduct 3. Photolysis of a benzene solution of 3 at 365 nm gives rise to the carbene dimer, namely the 1,2-bis(diisopropylamino)ethylene as a *cis*/*trans* mixture, along with white and red phosphorus. Under the same experimental conditions, but in the presence of excess 2,3-dimethyl-1,3-butadiene, the classical double Diels–Alder adduct of the triple-bonded diphosphorus P≡P was obtained along with the bis(phospholene) formally resulting from a double [4 + 1] reaction of the diene with the bis(phosphinidene) form of P_2_. A stepwise carbene–carbene exchange reaction also occurs between the monosubstituted aminocarbene of 3 and a cyclic (alkyl)(amino)carbene, possibly involving the transient formation of a diphosphorus analogue of a diazo compound.

## Abstract 2

The bis-monosubstituted aminocarbene-P_2_ adduct is a generator of P_2_, under the classical triple-bonded form (P≡P), but it also acts as a bis(phosphinidene) (P–P) synthetic equivalent.

## Graphical abstract

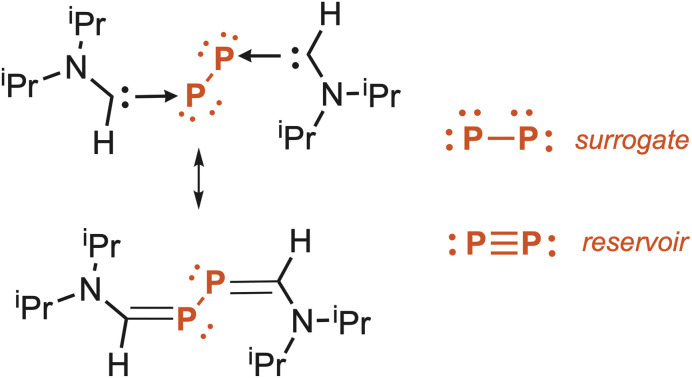

Elemental phosphorus exists in several allotropic forms. Red, violet and black phosphorus are polymeric, whereas white phosphorus is a tetra-atomic molecule. Unlike dinitrogen, discrete diphosphorus (P_2_) is unstable due to the high energy of the triple bond.^1^ P_2_ has been spectroscopically characterized both at high temperatures (>1100 K)^2^ and in matrices at a few K.^3^ P_2_ is also widely seen in the coordination sphere of transition metals.^4^ However, precursors able to generate P_2_ in solution and under mild conditions are highly desirable to make this species synthetically useful. This has been achieved by Cummins *et al.*^5^ and by Wolf and Goicoechea *et al.*^6^ who were able to induce Diels–Alder reactions between transient P_2_ and dienes, and by Scheer *et al.*^7^ who trapped P_2_ with a molybdenum complex (Fig. 1A). A decade ago, Robinson *et al.*,^8^ as well as our group,^9^ showed that compounds A and B can be viewed as diphosphorus stabilized by N-heterocyclic carbenes (NHCs)^10^ and cyclic (alkyl)(amino)carbenes (CAACs),^11^ respectively (Fig. 1B). Herein, we report the synthesis of a compound featuring a P_2_ fragment capped by two monosubstituted aminocarbenes and show that the diphosphorus unit can be transferred, exhibiting the behaviour of both triple-bonded diphosphorus (P≡P) and bis(phosphinidene) (P–P) (Fig. 1C).

**Fig. 1:**
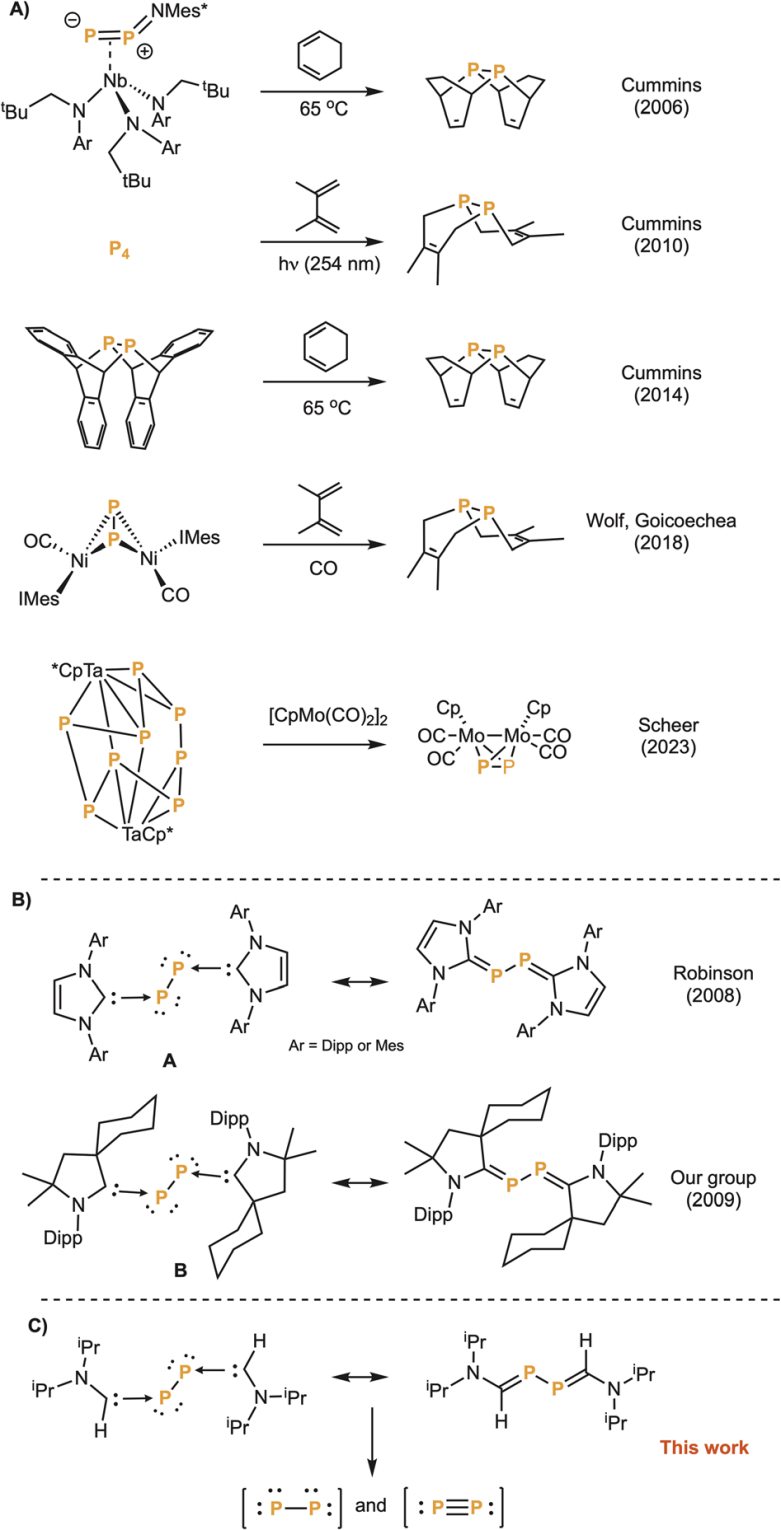
(A) Previously reported examples of generation and trapping of P_2_. (B) Bis(NHC) and bis(CAAC) adducts of P_2_. (C) Diphosphorus stabilized by monosubstituted aminocarbenes as triple-bonded diphosphorus and bis(phosphinidene) transfer agent.

Following our discovery that phosphaketenes are the direct precursor of phosphinidenes,^12^ we envisaged that the reaction of NaPCO^13^ with a haloiminium salt, followed by deprotonation and loss of CO would lead to an amino phosphaalkyne (R_2_N–C≡P), a class of compounds scarcely accessible by known methods.^14^ Instead, by reacting the *N*,*N*-diisopropyl bromoiminium bromide 1 with NaPCO(dioxane)_3.5_, we observed the surprising formation of 2, the structure of which was ascertained by a single crystal X-ray diffraction study (Fig. 2). We quickly realized that a decarbonylation of 2 would lead to a P_2_ fragment stabilized by two small monosubstituted aminocarbenes.

**Fig. 2:**
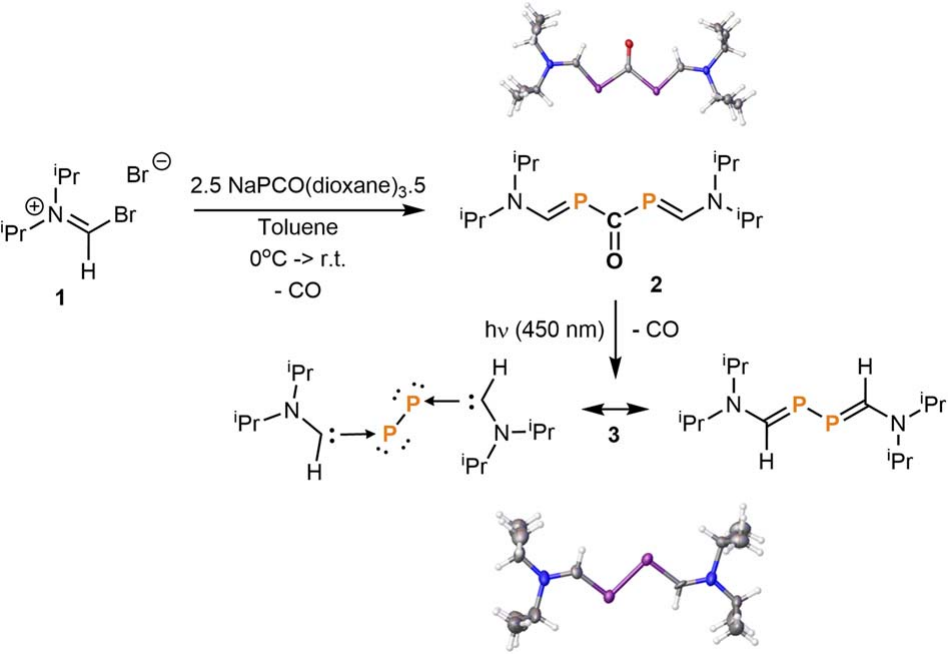
Synthesis of diphospha-urea 2 and subsequent photolytic decarbonylation under blue light irradiation to afford bis(carbene)P_2_ adduct 3.

Photolytic decarbonylation of diphospha-ureas [R_2_P(CO)PR_2_] has precedents,^15^ and gratifyingly, we found that the irradiation of a benzene solution of 2 at 450 nm (blue light) induced the elimination of CO quantitatively, affording the desired compound 3. The X-ray crystal structure of 3 reveals that the two carbene units are coplanar, whereas for the analogous compounds A and B, the bulky carbene units are forced to adopt a twisted conformation to accommodate the steric congestion. The PP bond distance in 3 (2.18 Å) is very similar to those observed for A (2.19 Å) and B (2.18 Å), while the C–P bond lengths in 3 (1.71 Å) are comparable to that of B (1.72 Å) but slightly shorter than in A (1.75 Å). The ^31^P NMR spectrum of 3 showed a downfield signal at +78.0 ppm akin to that of the bis(CAAC)P_2_ adduct B (+59.4 ppm), whereas Robinson's bis(NHC)P_2_ adduct A exhibits a considerably more shielded signal at −73.6 ppm. This is due to the weaker p-accepting ability of NHCs *versus* CAACs and monosubstituted aminocarbenes.^16^ These data collectively indicate that the diphosphabutadiene^17^ resonance structure is significant in 3 as it is in B.

Despite not having a strong bis(carbene)P_2_ character, we wondered if the carbene units of 3 could be released to generate P_2_. Upon heating 3 at 80 °C for 6 hours, no reaction was observed. However, upon irradiating a C_6_D_6_ solution of 3 (*λ*_max_ = 349 nm) with 365 nm light, we observed the formation of the carbene dimer, namely the 1,2-bis(diisopropylamino)ethylene 4 as a *cis*/*trans* mixture, along with P_4_ and a red precipitate indicative of red phosphorus (Scheme 1). This was the first evidence for the transient formation of P_2_, which is known to spontaneously dimerize and polymerize to white and red phosphorus, respectively.^18^ Based on this encouraging result, we investigated the possibility of intercepting P_2_ using a 1,3-diene – a reaction well-established in the literature.^5,6^ Irradiation of a THF solution of 3 at 365 nm in the presence of a large excess of 2,3-dimethyl-1,3-butadiene gave rise to phosphorus containing products 5 and 6 (2/1 ratio), as well as traces of 7, in addition to the carbene dimer 4 as a *cis*/*trans* mixture (Scheme 1). The major phosphorus compound 5 is the expected double [4 + 2] Diels–Alder adduct of the triple-bonded diphosphorus, the product observed by Cummins *et al.*^5^ and by Wolf and Goicoechea *et al.*^6^ More surprisingly, 6 formally results from two [4 + 1] cycloadditions of the diene with P_2_ acting as a bis(phosphinidene) (P–P), followed by insertion of the carbene dimer 4 into the PP bond. Note that the reaction of phosphinidenes and phosphinidene metal complexes with dienes giving the corresponding phospholenes have precedents,^19^ as well as the insertion of alkenes into diphosphines.^20^ We prepared the (bis)phospholene 7 according to a literature procedure,^21^ added the alkene 4 in THF, and after photolysis at 365 nm, compound 6 was obtained.

**Scheme 1:**
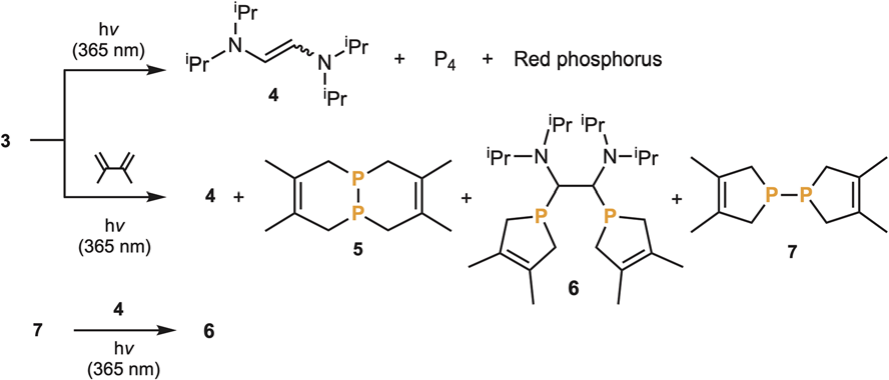
Irradiative cleavage of carbene fragments from 3 and trapping of diphosphorus with 2,3-dimethylbutadiene to give double Diels–Alder products 5 and bis(phospholene)-alkene adduct 6, with traces of 7. Independent synthesis of 6 through photolysis of a mixture of 7 and 4.

The formation of 6 and 7 is intriguing and suggests that the elimination of the two aminocarbenes 8 occurs stepwise. The first carbene elimination leads to the hitherto unknown diphosphorus analogue 9 of a diazo compound, which is trapped *via* a [4 + 1] cycloaddition with dimethylbutadiene giving 10. Then a second carbene elimination gives a phosphinidene, which also reacts with the diene affording 7 (Scheme 2).

**Scheme 2:**
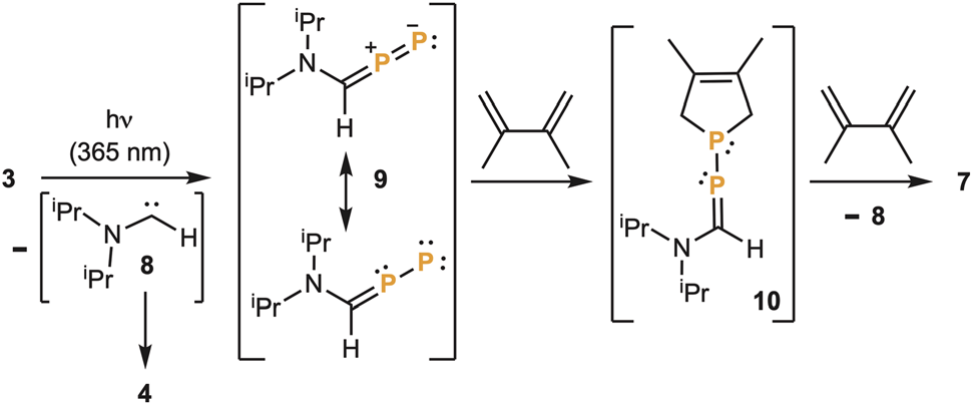
Proposed intermediates in the formation of 7, *via* stepwise elimination of carbene fragments from 3.

In 2016, we reported that a stable singlet phosphinidene^12^ quickly reacted with carbenes to give the corresponding adduct.^22^ Therefore, in an attempt to trap P_2_ as a (bis)phosphinidene, we added five equivalents of cyclic (alkyl)(amino)carbene 11,^11*a*^ to a THF solution of 3. To our surprise, without any irradiation, a clean reaction occurred giving rise to the bis(CAAC)P_2_ adduct 12,^9^ along with the mixed carbene dimer 13. Repeating the experiment, with only two equivalents of CAAC 11, we observed the formation of 14, along with 13, clearly demonstrating the stepwise nature of the reaction (Scheme 3). These carbene–carbene exchange reactions are similar to those observed with the stable phosphinidene, for which DFT calculations predicted an associative mechanism.^23^ The formation of the mixed carbene dimer 13 confirms the prediction. Note that no exchange reaction occurred with imidazole-2-ylidenes.

**Scheme 3:**
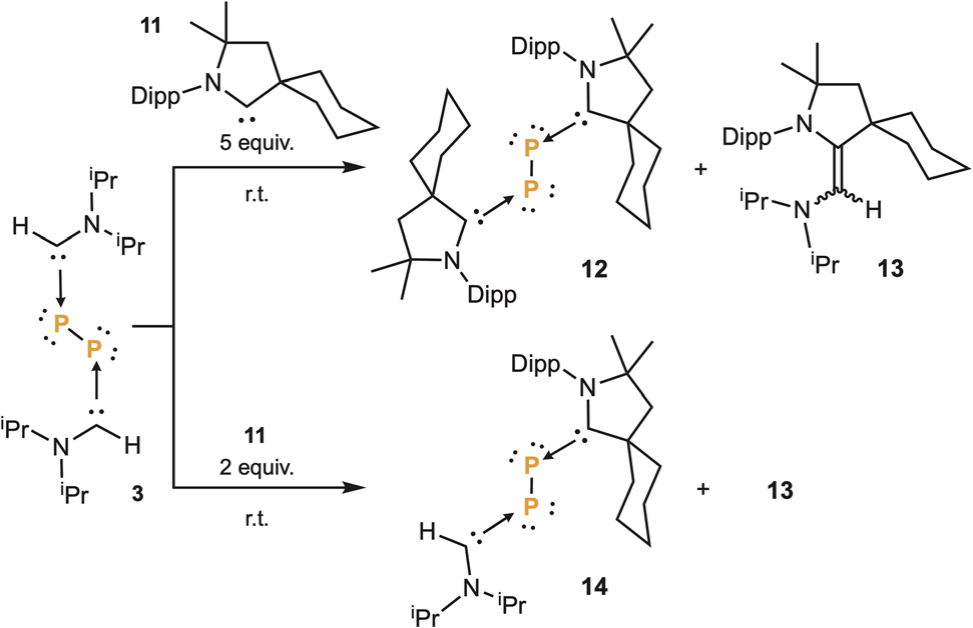
Double and mono substitution of aminocarbenes at diphosphorus with CAAC 11, affording 12 and 14, with concomitant formation of mixed carbene dimer 13.

## Conclusions

In summary, we disclose a new synthetic route to bis(carbene)P_2_ adducts. Under irradiation at 365 nm, the bis(monosubstituted aminocarbene)P_2_ adduct 3 is a generator of P_2_, under the classical triple-bonded form (P≡P), but 3 also acts as a bis(phosphinidene) (P–P) synthetic equivalent. These findings provide new insights into the stabilization and reactivity of diphosphorus species, offering promising avenues for future synthetic applications.

## Author contributions

J. Y. performed synthetic experiments. J. Y. and M. A. carried out the UV-vis studies. M. G. performed X-ray crystallographic analyses. G. B. supervised and designed the project. The manuscript was written by J. Y., M. A., and G. B.

## Conflicts of interest

There are no conflicts to declare.

## Supplementary Material

SC-015-D4SC05091F-s001

SC-015-D4SC05091F-s002

## Data Availability

All experimental procedures and characterization data can be found in the ESI.†
