# Supplementary material for: A carbene-stabilized diphosphorus: a triple-bonded diphosphorus (P[triple bond, length as m-dash]P) and a bis(phosphinidene) (P–P) transfer agent
Source: Chem Sci. 2024 Sep 2;15(38):15713–6. doi: 10.1039/d4sc05091f (PMC11379058; doi:10.1039/d4sc05091f)
Supplement: SC-015-D4SC05091F-s001 [file SC-015-D4SC05091F-s001.pdf]

Supplementary Information (SI) for Chemical Science.
This journal is © The Royal Society of Chemistry 2024

Supporting Information

A Carbene-Stabilized Diphosphorus: A Triple-Bonded Diphosphorus (P≡P) and (Bis)phosphinidene (P-P) Transfer Agent

Joseph S Yoon, Mehdi Abdellaoui, Milan Gembicky, and Guy Bertrand*

UCSD-CNRS Joint Research Laboratory (IRL3555), Department of Chemistry and Biochemistry, University of California San Diego, La Jolla, CA 92093-0358 (USA)

Table of contents

I. General information S2
II. Experimental procedures S3
III. NMR spectra S7
IV. UV-Visible analysis S24
V. X-ray crystallographic data S26
VI. References S33

I. General information

All reactions were performed under an atmosphere of argon by using standard Schlenk or dry box techniques. Carbene **11** was synthesized according to the previous literature.[i] The bis(phospholine) **7** was independently synthesized from known methods[ii] with new characterization data in this document (see NMR and X-ray sections).

Materials: Solvents were dried by distillation over sodium (benzene, toluene, diethyl ether, tetrahydrofuran, pentanes) or calcium hydride (dichloromethane, chloroform, hexanes). 2,3-dimethyl-1,3-butadiene was freshly distilled over sodium borohydride and stored in a dry-box freezer over 3 Å molecular sieves. All other reagents were purchased from commercial sources and used without further purification unless otherwise noted.

Instrumentation: NMR spectra were recorded on a Varian INOVA 500 MHz or JEOL 400 MHz spectrometer. Chemical shift values for ^{1}H are referenced to the residual protio-solvent (^{1}H) resonance of C_6D_6 (δ: 7.16), $CDCl_3$ (δ: 7.26), THF-d_8 (δ:3.58, 1.73). Chemical shift values for ^{13}C are referenced to the solvent (^{13}C) resonance of C_6D_6 (δ: 128.1), $CDCl_3$ (δ: 77.2), THF-d_8 (δ:67.6, 25.4). Chemical shifts are quoted in δ (ppm) and coupling constants in J (Hz) and multiplicities are abbreviated as follows: s = singlet, d = doublet, t = triplet, sept = septet, m = multiplet, brs = broad signal.

High-resolution mass spectrometry measurements were performed at the UC San Diego Mass Spectrometry Laboratory on an Agilent 6230 Accurate-Mass TOFMS spectrometer. Single crystal X-ray diffraction data were collected on Bruker Apex diffractometers using Mo-Kα radiation (λ = 0.71073 Å) or Cu-Kα radiation (λ = 1.54178 Å) at the UC San Diego Crystallography Facility. Absorption spectra were recorded on a JASCO V-630 spectrophotometer at ambient temperature

II. Experimental procedures

Preparation of 1-Br and 1-OTf

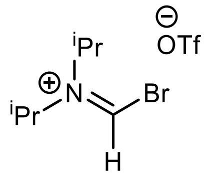

1-OTf

Preparation of bromide salt: A solution of *N*,*N*-diisopropyl formamide (3.0 mL; 10.35 mmol) in toluene (100 mL) was cooled to -78 °C. Providing a gentle flow of argon, oxalyl bromide (2.9 mL; 10.35 mmol) was slowly added over the course of 1 minute with vigorous stirring. The cold bath was removed, and a snow-white solid precipitated within several minutes. After 2 hours, the slurry was filtered leaving behind a snow-white solid. The bromide salt was washed three times with diethyl ether (about 20 mL per wash) and dried under vacuum to afford **1-Br** (5.75g; quantitative) which was used without further purification or characterization.

Counter anion exchange to triflate: **1-Br** (3.18g; 11.7 mmol) was resuspended in dichloromethane and cooled to -78 °C followed by the slow addition of trimethylsilyl triflate (4.2 mL; 23.3 mmol). The cold bath was removed, and the reaction proceeded for 2 hours at room temperature. The volatiles were removed under vacuum. The resulting brown residue was triturated with benzene (25 mL), inducing the precipitation of a tan solid. The product was filtered, washed three times with hexane (about 10 mL per wash), and dried to afford **1-OTf** (3.60g; 90.1% yield) as an off-white powder.

^{1}H NMR (500 MHz, $CDCl_3$) δ = 10.46 (s, 1H), 5.06 (sept, $^{3}J_{HH}$ = 6.7 Hz, 1H, CH(CH_3)$_2$), 4.63 (sept, $^{3}J_{HH}$ = 6.7 Hz, 1H, CH(CH_3)$_2$), 1.62 (d, $^{3}J_{HH}$ = 6.7 Hz, 6H, CH(CH_3)$_2$), 1.56 (d, $^{3}J_{HH}$ = 6.7 Hz, 6H, CH(CH_3)$_2$).

^{13}C{^{1}H} NMR (126 MHz, $CDCl_3$) δ = 162.28, 120.65 (q, $^{1}J_{CF}$ = 320.0 Hz), 62.07, 61.67, 23.15, 19.17.

^{19}F NMR (376 MHz, $CDCl_3$) δ = -78.17.

Preparation of 2

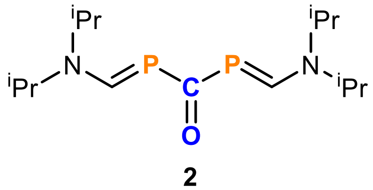

2

From **1-Br**: A solid mixture of **1-Br** (1.1 g; 4.03 mmol) and [Na(PCO)(dioxane)$_{3.5}$] (3.9 g; 9.91 mmol) was cooled to 0 °C and wrapped with aluminum foil. Under a gentle flow of argon, toluene (45 mL) was added. A vent needle to an oil bubbler was attached to prevent buildup of pressure, and the reaction was allowed to warm to room temperature overnight with stirring. The solution was filtered, and the product was extracted twice with DCM, and finally once more with toluene. Volatiles were evaporated under vacuum at

35 °C. The resulting orange solid was washed three times with cold diethyl ether and dried under vacuum to afford **2** (0.94 g; 74% yield).

From **1-OTf**: The above procedure was repeated starting with a solid mixture of **1-OTf** (0.8 g; 2.34 mmol) and [Na(PCO)(dioxane)$_{3.5}$] (2.27 g; 5.61 mmol) to afford **2** (0.22 g; 60% yield).

^{1}H NMR (500 MHz, C_6D_6) δ = 10.73 (t, $^{2}J_{HP}$ = 7.0 Hz, 2H, NC*H*P), 4.00 (m, 2H, C*H*(CH_3)$_2$), 2.89 (sept, $^{3}J_{HH}$ = 6.4 Hz, 2H, C*H*(CH_3)$_2$), 0.79 (d, $^{3}J_{HH}$ = 6.7 Hz, 12Hz, CH(C*H*$_3$)$_2$), 0.74 (d, $^{3}J_{HH}$ = 6.7 Hz, 12H, CH(C*H*$_3$)$_2$).

^{13}C{^{1}H} NMR (126 MHz, C_6D_6) δ = 236.4 (t, $^{1}J_{CP}$ = 81.6 Hz, P*C*(O)P), 187.3 (dd, $^{1}J_{CP}$ = 21.6 Hz, $^{3}J_{CP}$ = 14.8 Hz, N*C*HP), 55.8 (t, $^{3}J_{CP}$ = 8.5 Hz, *C*H(CH_3)$_2$), 48.9 (*C*H(CH_3)$_2$), 23.1 (CH(*C*H$_3$)$_2$), 18.6 (CH(*C*H$_3$)$_2$).

^{31}P{^{1}H} NMR (121 MHz, $CDCl_3$) δ = 84.0 (s).

^{31}P NMR (121 MHz, $CDCl_3$) δ = 84.0 (broad s).

HRMS (ESI+): calcd. for **2** $[M+H]^+$ $[C_{15}H_{31}N_2OP_2]^+$ m/z: 317.1906, found 317.1909.

Preparation of 3

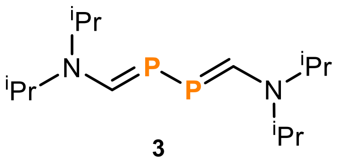

3

In a skinny pressure-sealed Schlenk flask, **2** (90 mg; 0.285 mmol) was dissolved in THF (15 mL). The resulting yellow solution was irradiated for 1 hour with 450 nm light while maintaining an internal temperature between 35-40 °C using a fan. The volatiles were removed under vacuum. The resulting orange solids were resuspended in pentane and cooled down to -35 °C. The product was filtered, washed with cold pentane, and dried under vacuum to give **3** (62 mg; 76% yield).

NOTE: For each experiment, the progression of conversion from **2** to **3** was monitored by ^{31}P NMR. The typical time required for full conversion varied by experiment, ranging between 50 to 70 minutes.

^{1}H NMR (400 MHz, THF-*d$_8$*) δ = 8.97 (s, 2H, NC*H*P), 3.99 (broad, 4H, C*H*(CH_3)$_2$), 1.17 (d, $^{3}J_{HH}$ = 6.8 Hz, 24H, CH(C*H*$_3$)$_2$).

^{13}C{^{1}H} NMR (101 MHz, THF-*d$_8$*) δ = 182.5 (dd, $^{1}J_{CP}$ = 12.6 Hz, $^{2}J_{CP}$ = 4.2 Hz, N*C*HP), 50.0 (*C*H(CH_3)$_2$), 21.5 (CH(*C*H$_3$)$_2$).

^{31}P{^{1}H} NMR (162 MHz, THF-*d$_8$*) δ = 79.9 (s).

^{31}P NMR (162 MHz, THF-*d$_8$*) δ = 79.9 (s).

HRMS (ESI+): calcd. For **3** $[M+H]^+$ $[C_{14}H_{31}N_2P_2]^+$ m/z: 289.1957, found 289.1957.

Photochemical degradation of 3

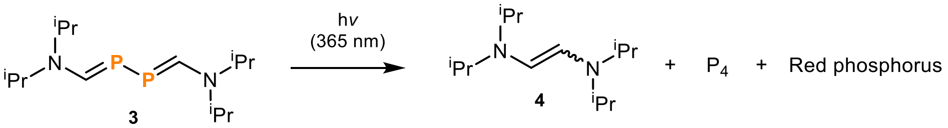

In a J-young NMR tube, **2** (53 mg; 0.17 mmol) was dissolved in C_6D_6 (0.6 mL) and irradiated with 450 nm light until full conversion to **3** was observed by ^{31}P NMR. This solution was then irradiated with 365 nm light overnight, resulting in the precipitation of a red solid. The resulting ^{1}H and ^{13}C NMR spectra confirmed the formation of carbene dimer, 1,2-bis(diisopropylamino)ethylene, as a *cis*/*trans* mixture (see NMR spectra section) accompanied by the disappearance of the starting material signals by ^{31}P NMR and the appearance of a singlet at -520 ppm (P_4). The identity of 1,2-bis(diisopropylamino)ethylene was also confirmed by mass spectroscopic analysis (calcd. For **4** $[M+H]^+$ $[C_{14}H_{31}N_2]^+$ m/z: 227.2482, found 227.2478.)

Formation of 4, 5, and 6

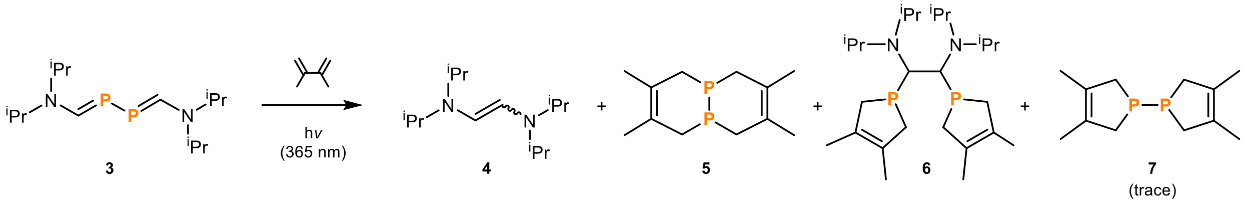

In a skinny pressure-sealed flask, **3** (75 mg; 0.26 mmol) was dissolved in a 1:1 (v/v) solution of THF and 2,3-dimethyl-1,3-butadiene (5 mL). With vigorous stirring, the solution was irradiated with 365 nm light overnight, while maintaining a temperature between 35-40 ºC in the photolysis chamber using a fan. Volatiles were removed under vacuum at room temperature. Using a minimal amount of THF, the mixture was transferred to a sublimator, where the volatiles were slowly removed under vacuum at room temperature. A water-cooled (0 ºC) cold finger was attached, and the mixture was heated to 110 ºC under strong vacuum, resulting in the deposition of white solids onto the cold finger. Inside a glovebox, the solids were extracted from the cold finger with pentane. The product was dried under vacuum, leaving behind a mixture of **4**, **5**, and **6**, along with a trace of **7** (calc. for **5** $[M+H]^+$ $[C_{12}H_{21}P_2]^+$ m/z: 227.1113, found 227.1115; calc. for **6** $[M+H]^+$ $[C_{26}H_{51}N_2P_2]^+$ m/z: 453.3522, found 453.3528).

Reaction of 4 and 7 to form 6

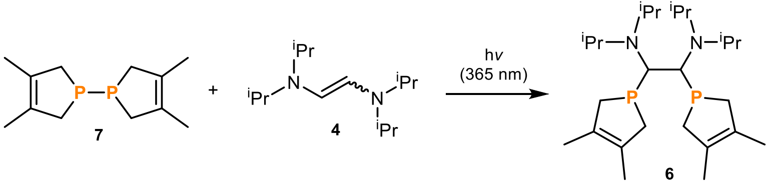

[Note: Compound **4** was prepared by the photolytic degradation of **3** with 365 nm light]. Solids **4** (ca. 1 mmol) and **7** (170 mg; 0.75 mmol) were placed in a pressure-sealed flask and dissolved in 15 mL THF

showing the bis(phospholine) ^{31}P NMR shift of -41.9 ppm. The contents were irradiated with 365 nm light overnight, resulting in the full conversion to **6** with a ^{31}P NMR shift of -48.6 ppm (see NMR section).

Synthesis of 12, 13, and 14

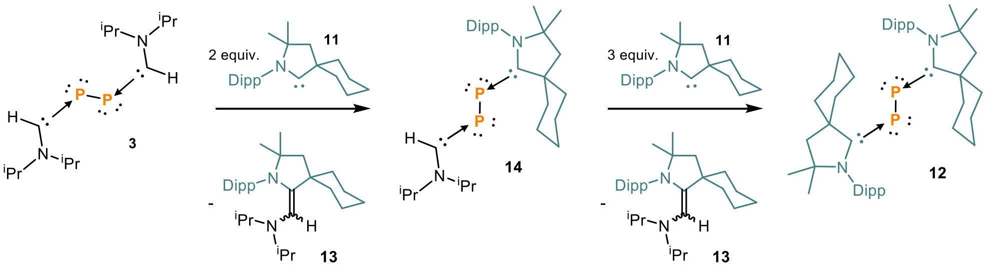

A 0.20 M solution of carbene **11** in THF (1.0 mL; 0.20 mmol) was added to **3** (30 mg; 0.10 mmol) in a J-young NMR tube. The reaction was monitored by ^{31}P NMR. After 2 days, **3** had been consumed and one major phosphorus containing species **14** was observed by NMR (see NMR spectra section). To this was added another aliquot of the 0.20 M solution of **11** in THF (1.5 mL; 0.30 mmol), giving quantitative conversion to **12** by ^{31}P NMR. Volatiles were removed and bright orange solids were precipitated out of cold ether. The orange solid was collected and washed with cold ether and dried under vacuum to give **12** (29 mg; 41% yield) (calc. for **12** $[M+H]^+$ $[C_{46}H_{71}N_2P_2]^+$ m/z: 713.5087, found 713.5085) (see NMR spectra section). The filtrates were combined and recrystallized by evaporation at -33 °C to give colorless cubic crystals, which were washed with minimal cold pentane to give **13** (28 mg; 32% yield) (calc. for **13** $[M+H]^+$ $[C_{30}H_{51}N_2]^+$ m/z: 439.4047, found 439.4050) (see NMR spectra section).

III. NMR spectra

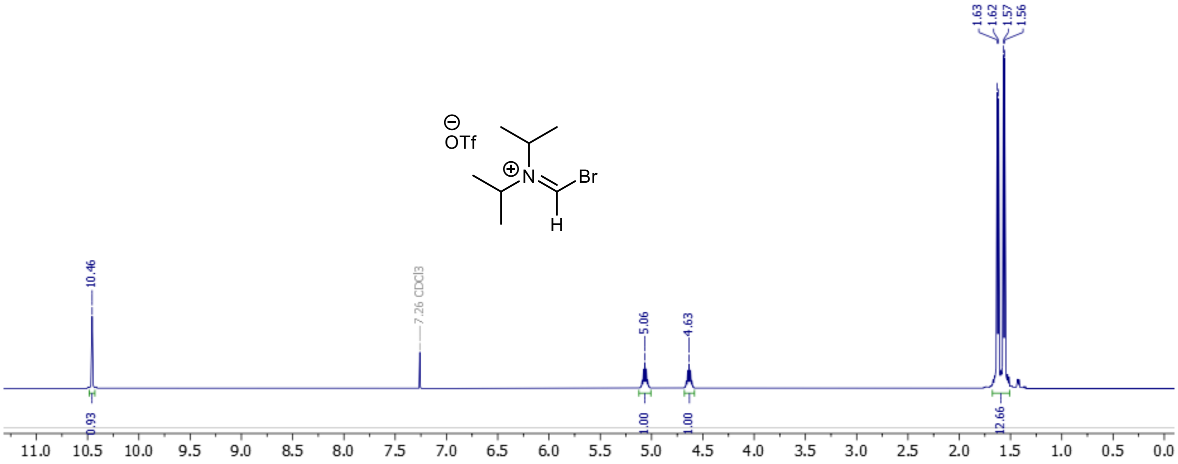

Figure S1. ^{1}H NMR (500 MHz) spectrum of **1-OTf** in $CDCl_3$.

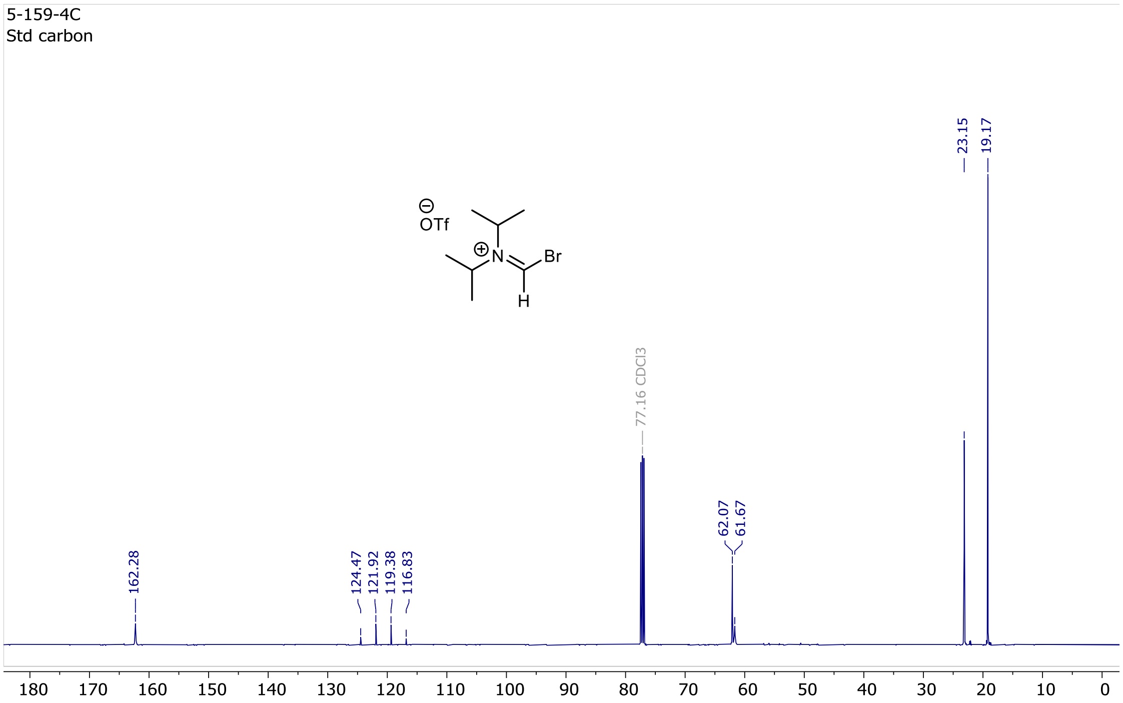

Figure S2. ^{13}C{^{1}H} NMR (126 MHz) spectrum of **1-OTf** in $CDCl_3$.

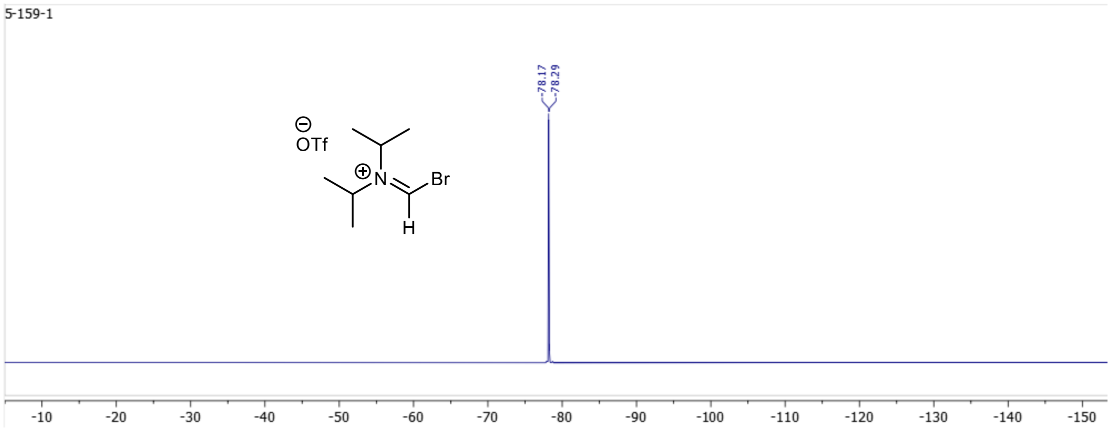

Figure S3. ^{19}F NMR (376 MHz) spectrum of **1-OTf** in $CDCl_3$.

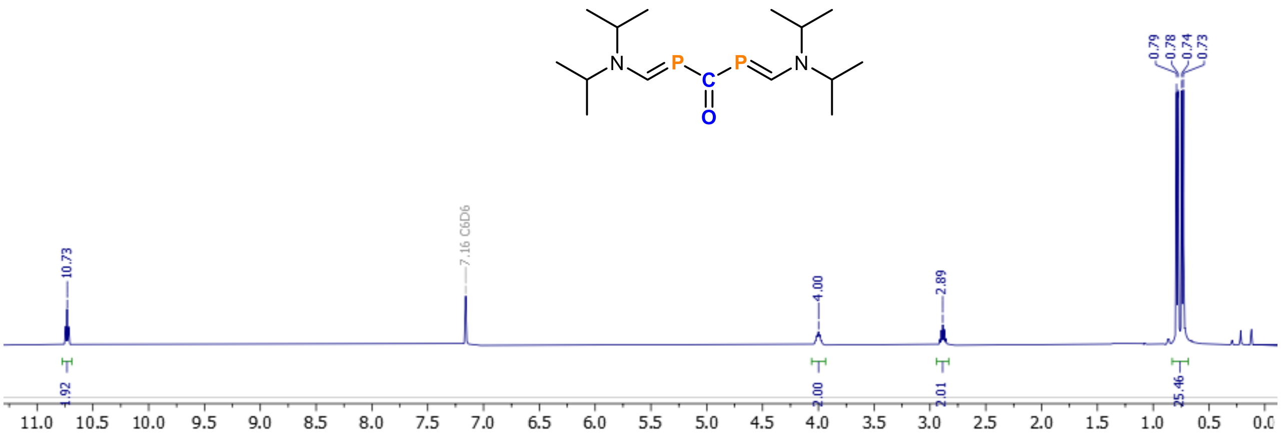

Figure S4. ^{1}H NMR (500 MHz) spectrum of **2** in C_6D_6.

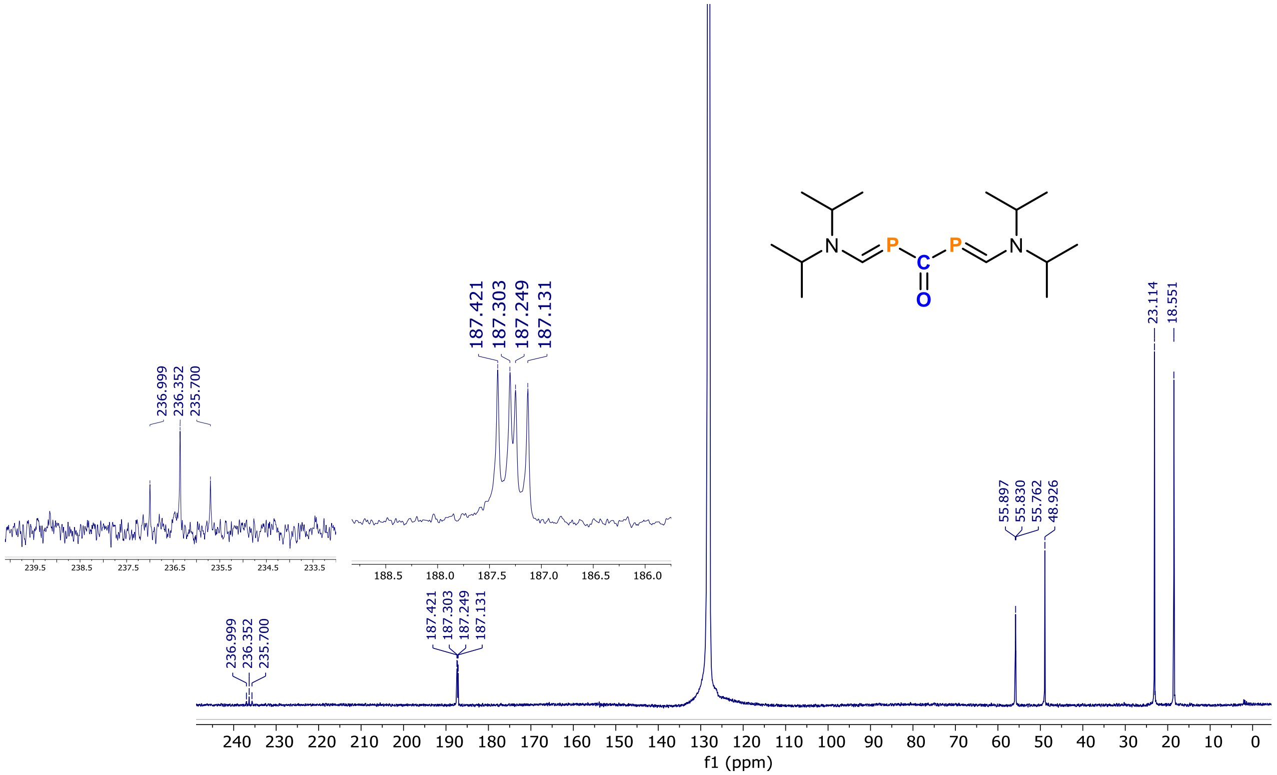

Figure S5. $^{13}C\{^{1}H\}$ NMR (126 MHz) spectrum of **2** in C_6D_6.

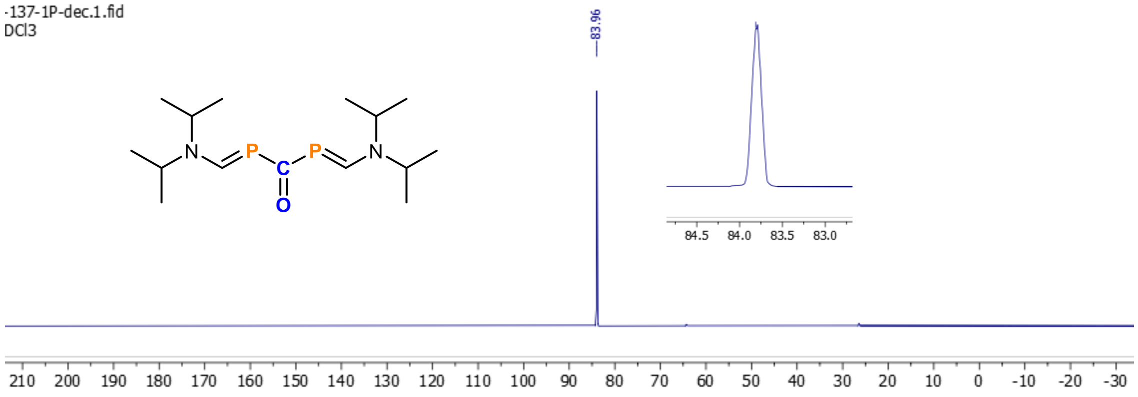

Figure S6. $^{31}P\{^{1}H\}$ and ^{31}P (inset) NMR (121 MHz) spectrum of **2** in $CDCl_3$.

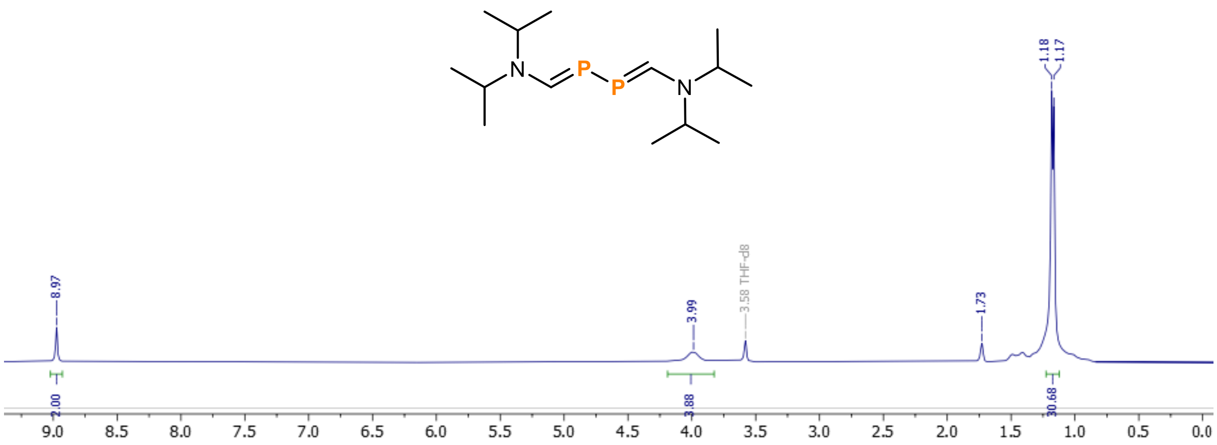

Figure S7. ^{1}H NMR (400 MHz) spectrum of **3** in THF-*d*.

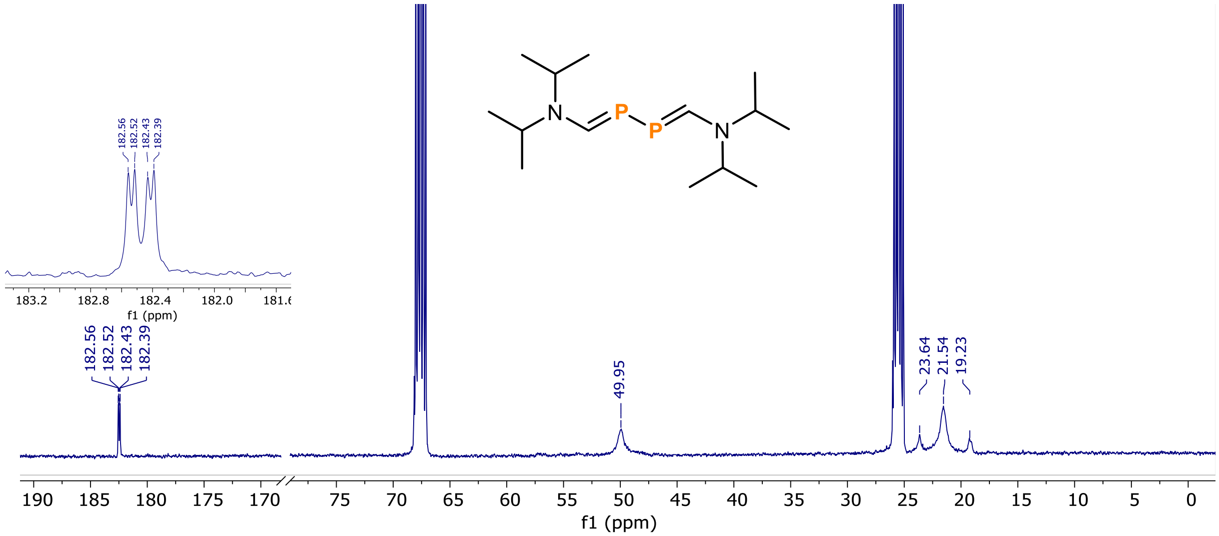

Figure S8. ^{13}C{^{1}H} NMR (101 MHz) spectrum of **3** in THF-d_8.

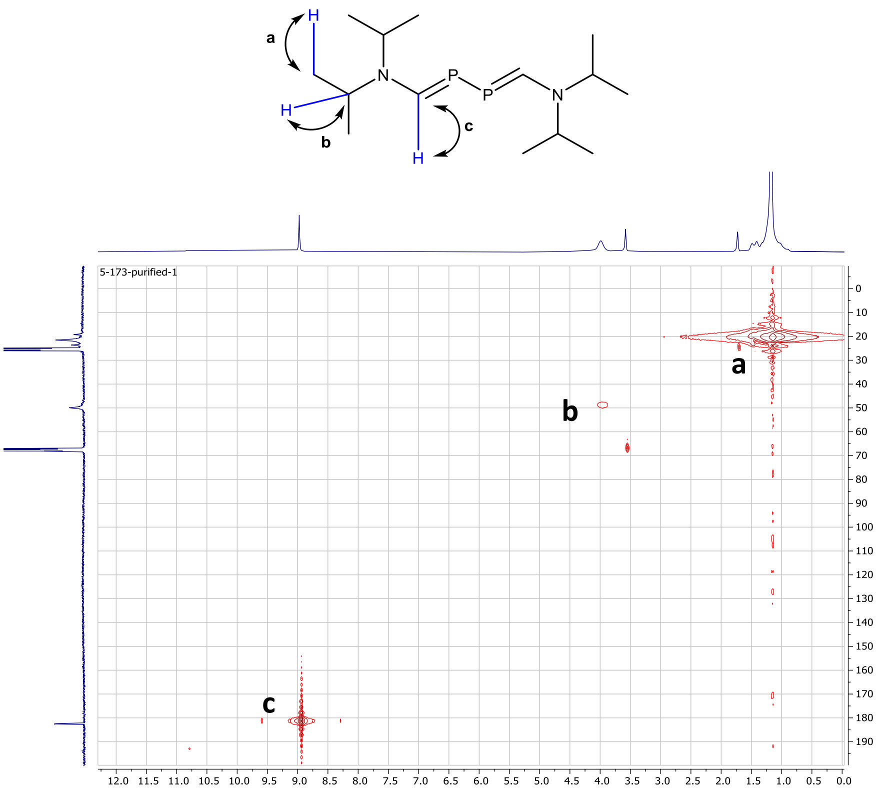

Figure S9. HMQC (400 MHz) spectrum of **3** in THF-d_8.

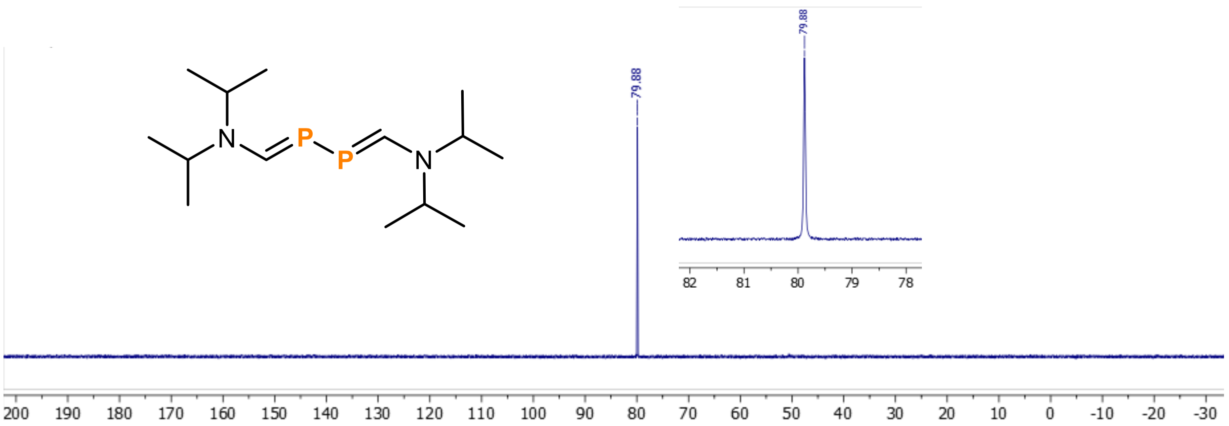

Figure S10. $^{31}P\{^{1}H\}$ and ^{31}P (inset) NMR (162 MHz) spectrum of **3** in THF-d_8.

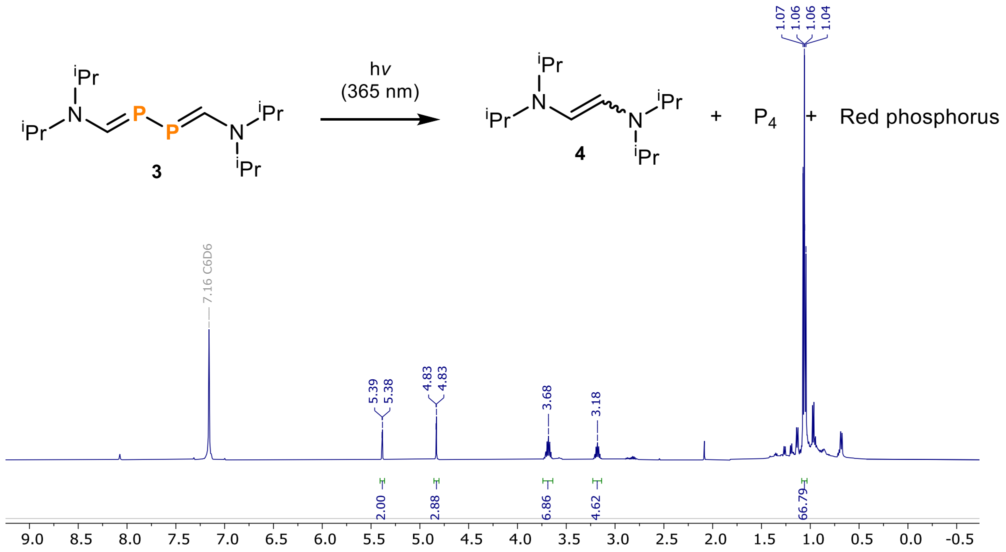

Figure S11. ^{1}H NMR (400 MHz) spectrum in C_6D_6 of **4**, via overnight treatment of **3** with 365 nm light.

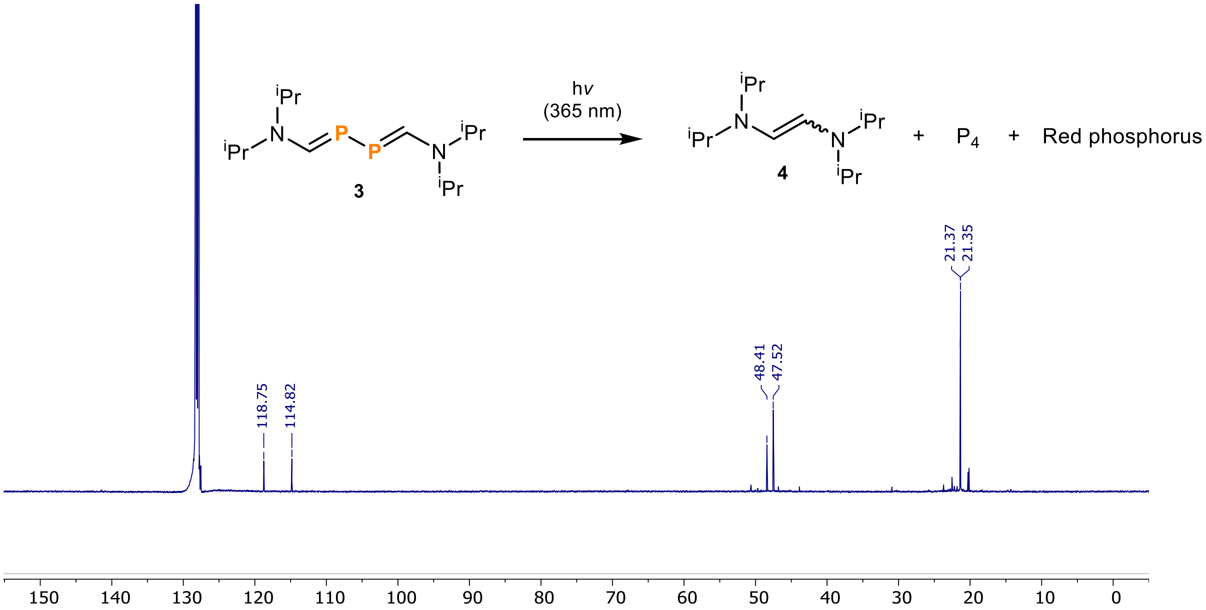

Figure S12. $^{13}C\{^1H\}$ NMR (126 MHz) spectrum in C_6D_6 of **4**, via overnight treatment of **3** with 365 nm light.

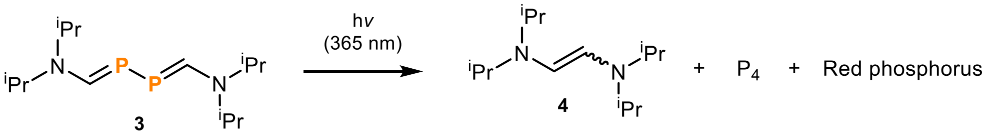

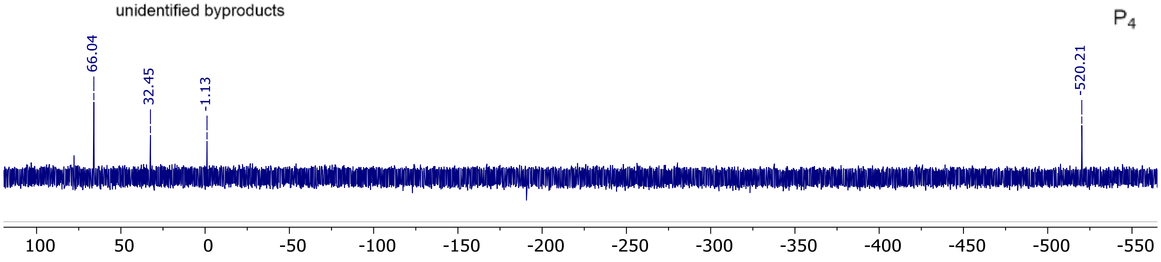

Figure S13. $^{31}P\{^1H\}$ NMR (162 MHz) spectrum in C_6D_6 after overnight treatment of **3** with 365 nm light.

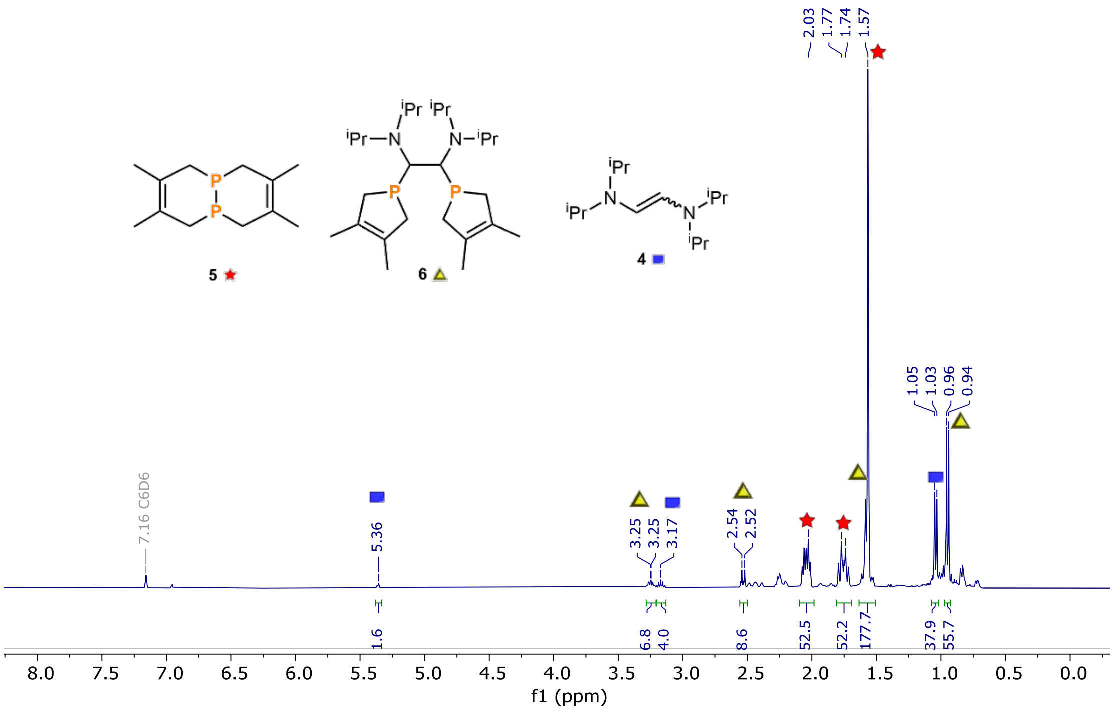

Figure S14. ^1H NMR (500 MHz) spectrum of **4**, **5**, and **6** in C_6D_6.

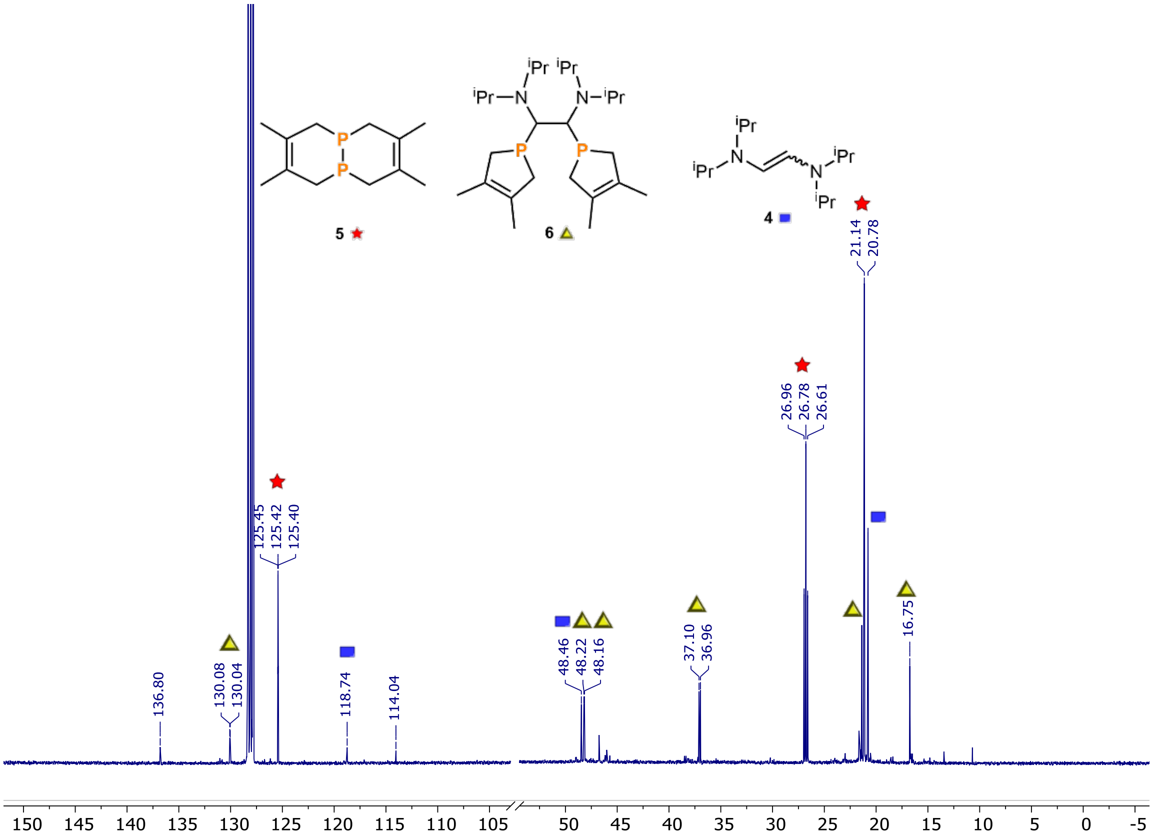

Figure S15. $^{13}C\{^1H\}$ NMR (101 MHz) spectrum of **4**, **5**, and **6** in C_6D_6.

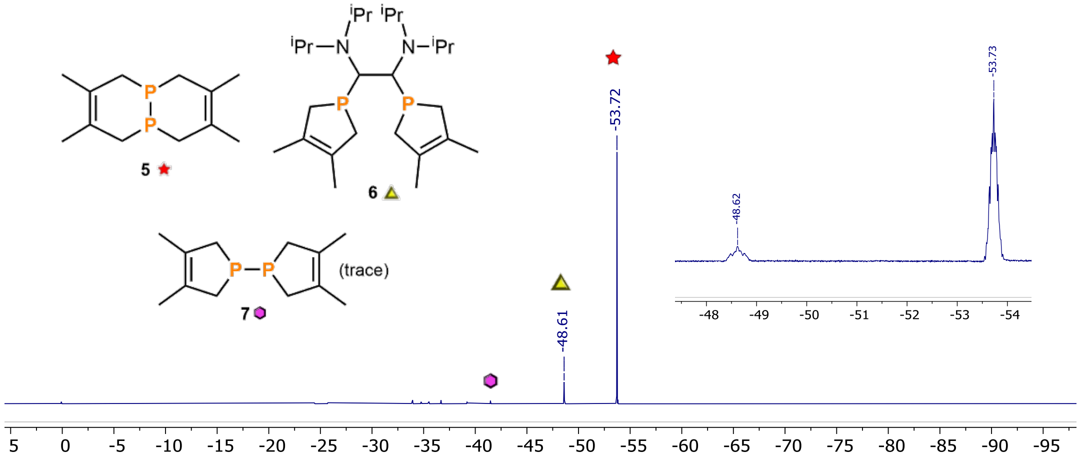

Figure S16. $^{31}P\{^1H\}$ and ^{31}P (inset) NMR (162 MHz) spectrum of **5**, **6**, and **7** in C_6D_6.

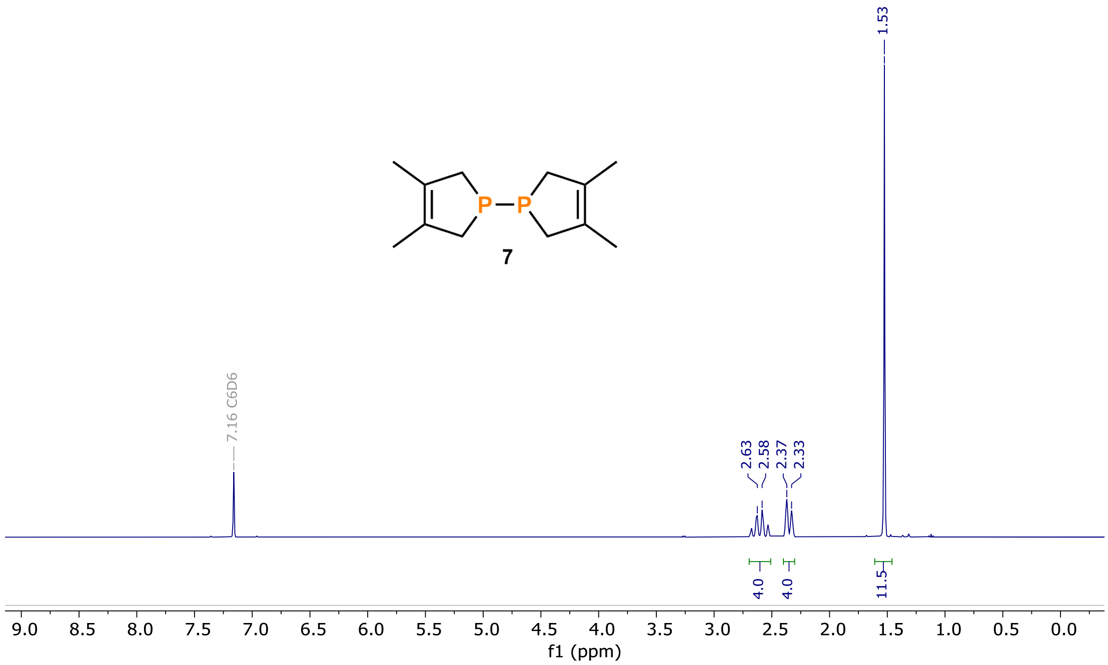

Figure S17. ^{1}H NMR (400 MHz) spectrum of **7** in C_6D_6.

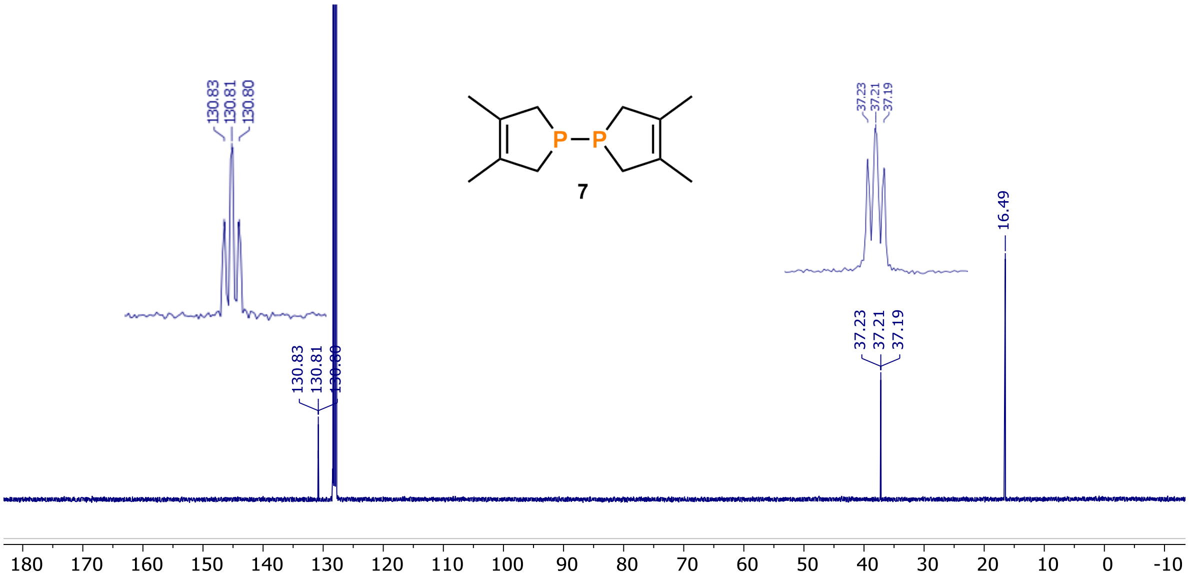

Figure S18. $^{13}C\{^{1}H\}$ NMR (101 MHz) spectrum of **7** in C_6D_6.

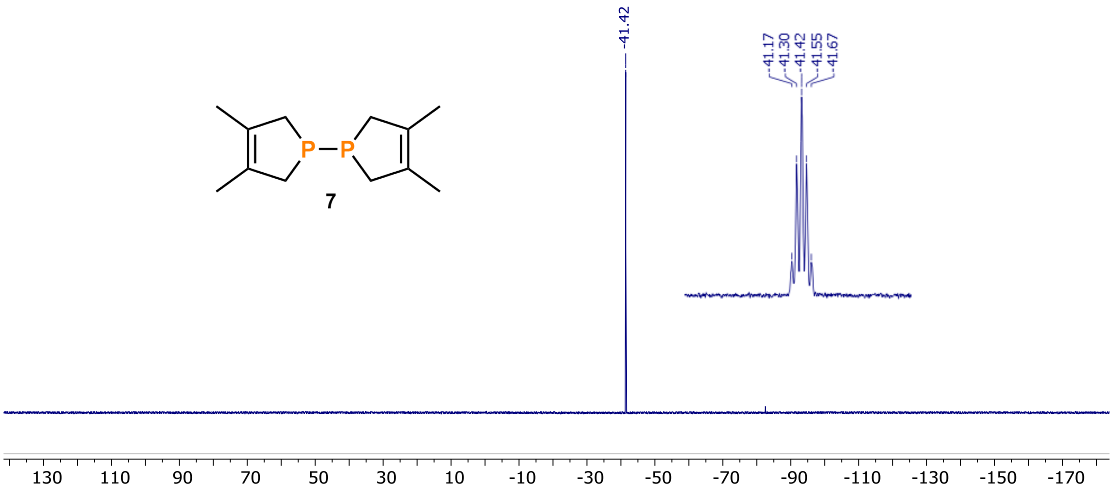

Figure S19. $^{31}P\{^{1}H\}$ and ^{31}P (inset) NMR (162 MHz) spectrum of **7** in C_6D_6.

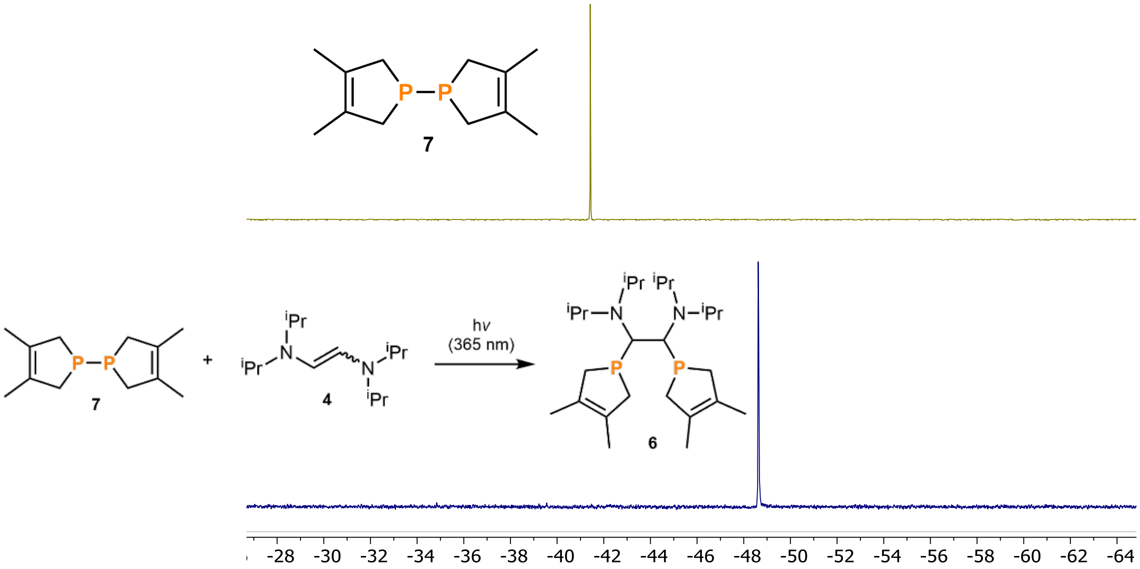

Figure S20. $^{31}P\{^{1}H\}$ NMR (162 MHz) spectra in THF-d8 of **6** (top) and the reaction of **7** with **4** to form **6** (bottom).

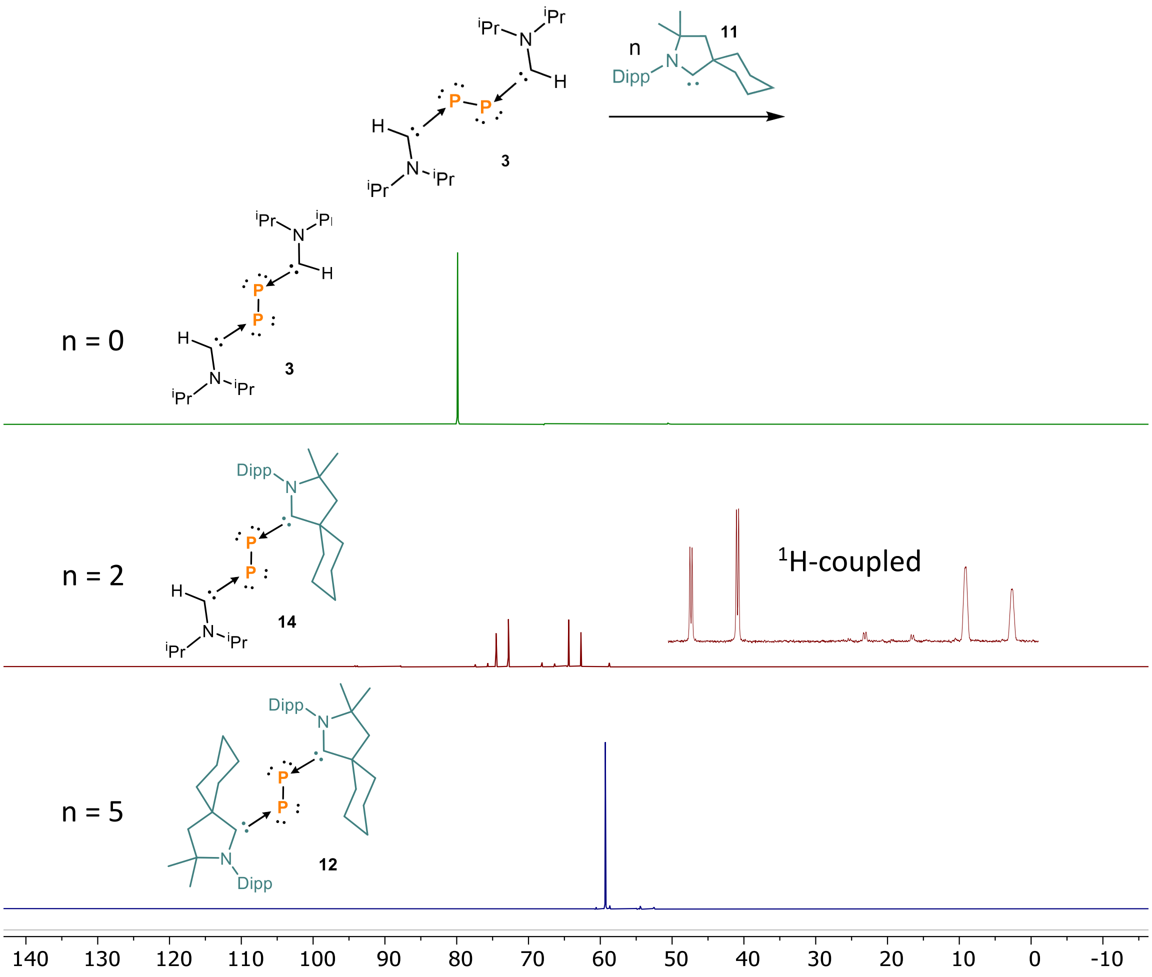

Figure S21. $^{31}P\{^{1}H\}$ NMR (162 MHz) spectra in THF-d_8 of **3** (green), with 2 equivalents of **11** (red), and with 5 equivalents of **11** (blue).

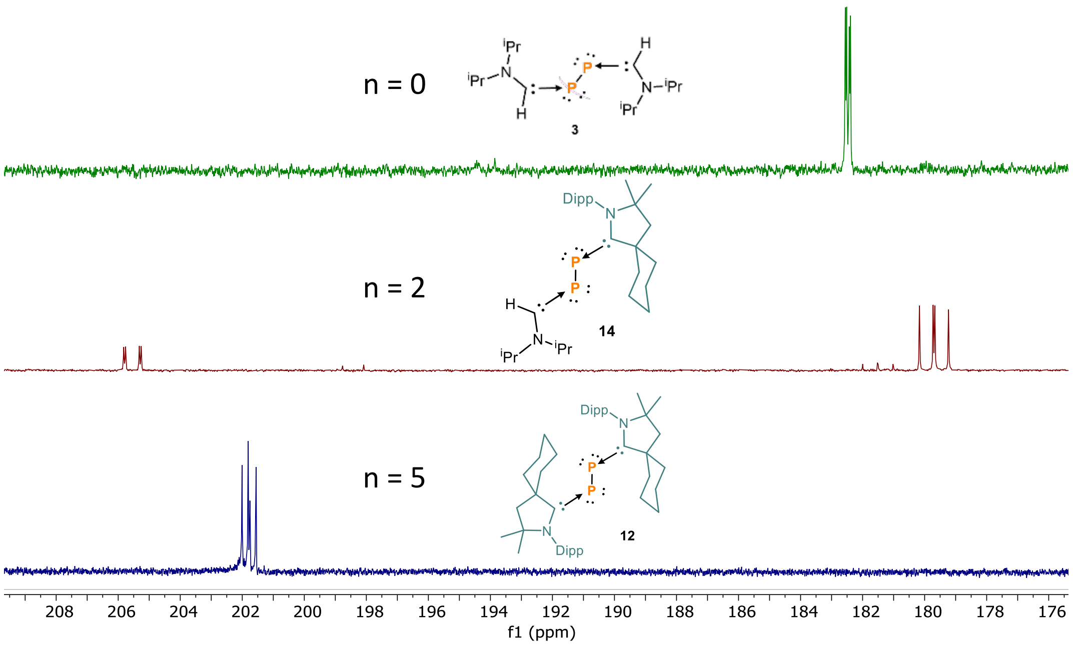

Figure S22. Carbene region of $^{13}C\{^1H\}$ NMR (126 MHz) spectra in THF-d_8 of **3** (green), with 2 equivalents of **11** (red), and with 5 equivalents of **11** (blue).

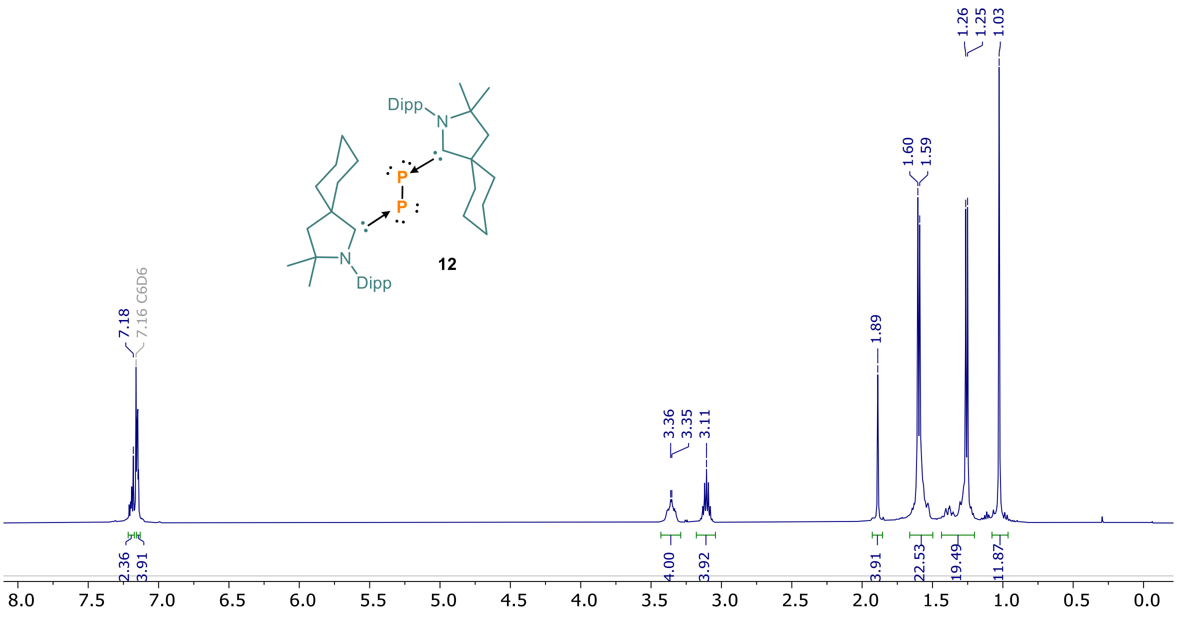

Figure S23. ^1H NMR (500 MHz) spectrum of **12** in C_6D_6.

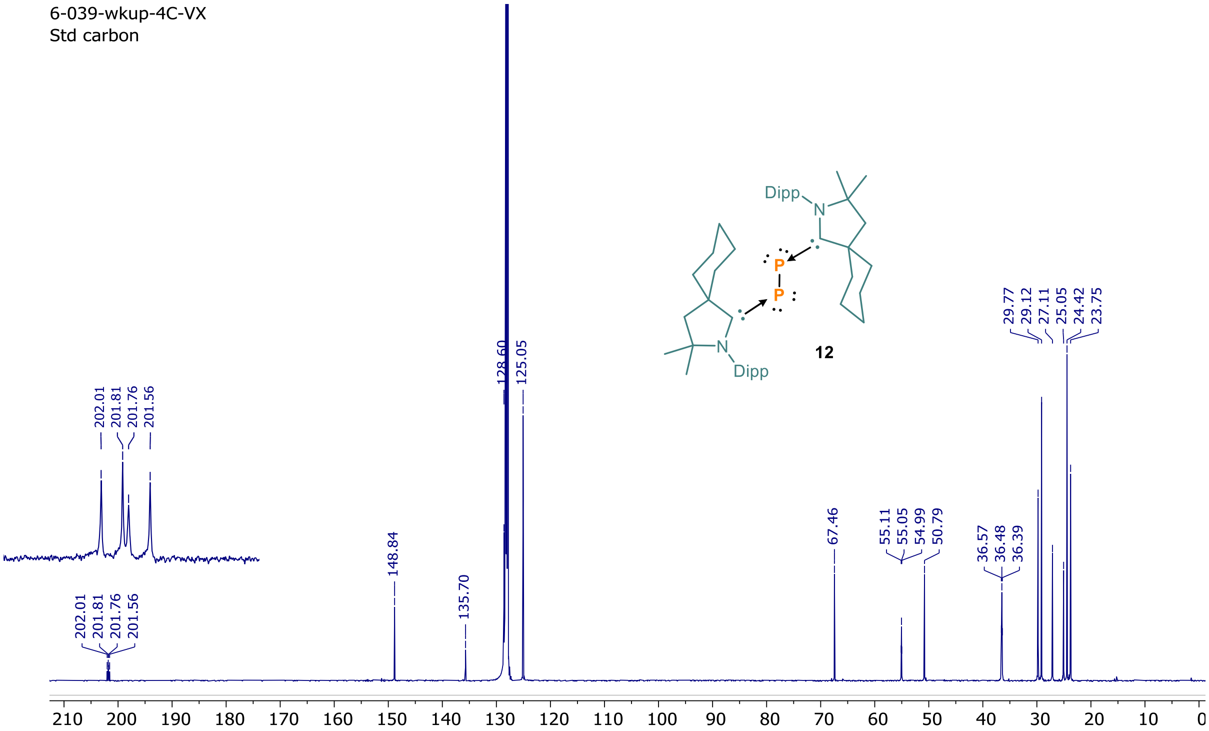

Figure S24. $^{13}C\{^{1}H\}$ NMR (126 MHz) spectrum of **12** in C_6D_6.

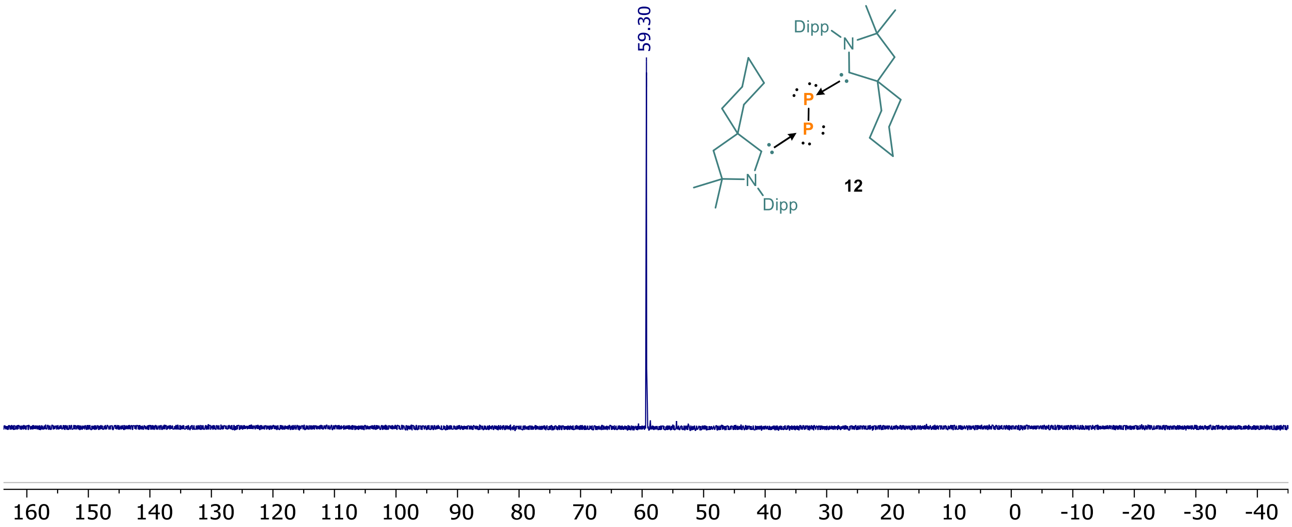

Figure S25. $^{31}P\{^{1}H\}$ NMR (162 MHz) spectrum of **12** in C_6D_6.

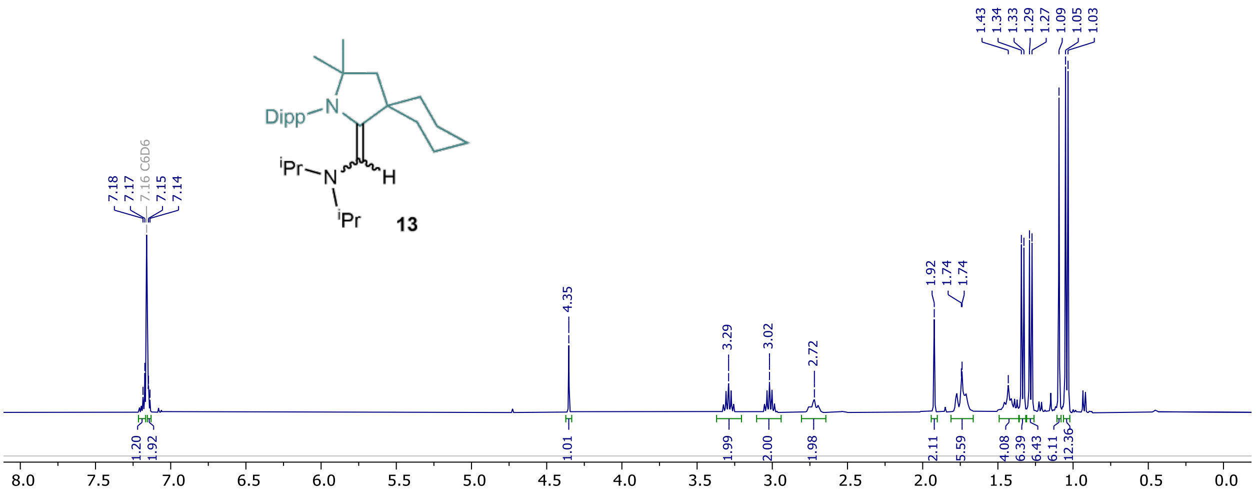

Figure S26. ^{1}H NMR (400 MHz) spectrum of **13** in C_6D_6.

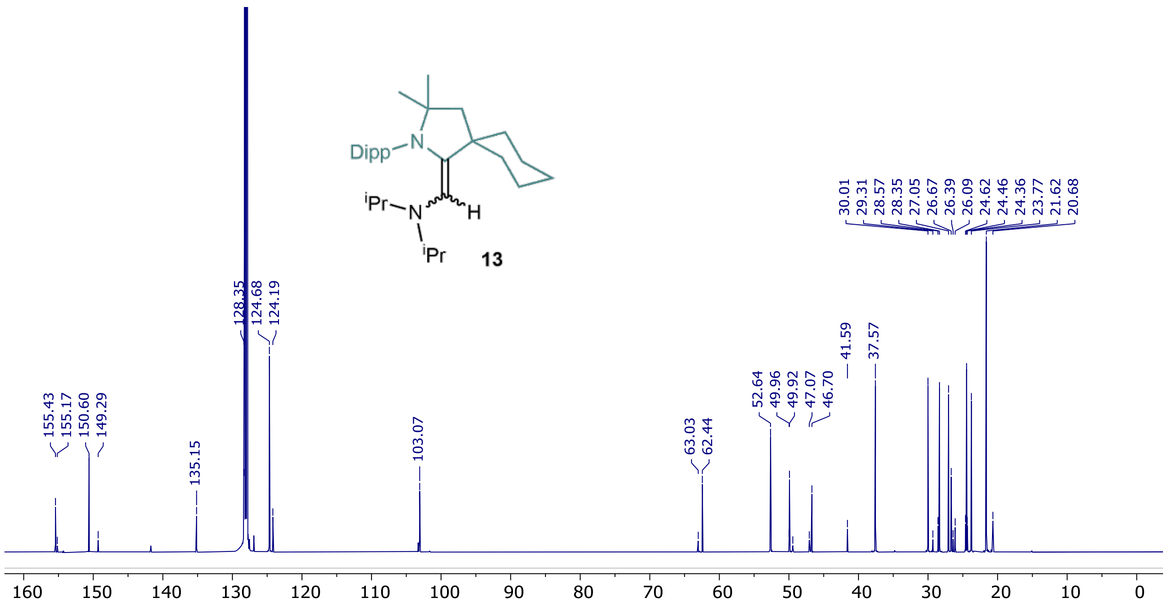

Figure S27. $^{13}C\{^{1}H\}$ NMR (126 MHz) spectrum of **13** in C_6D_6.

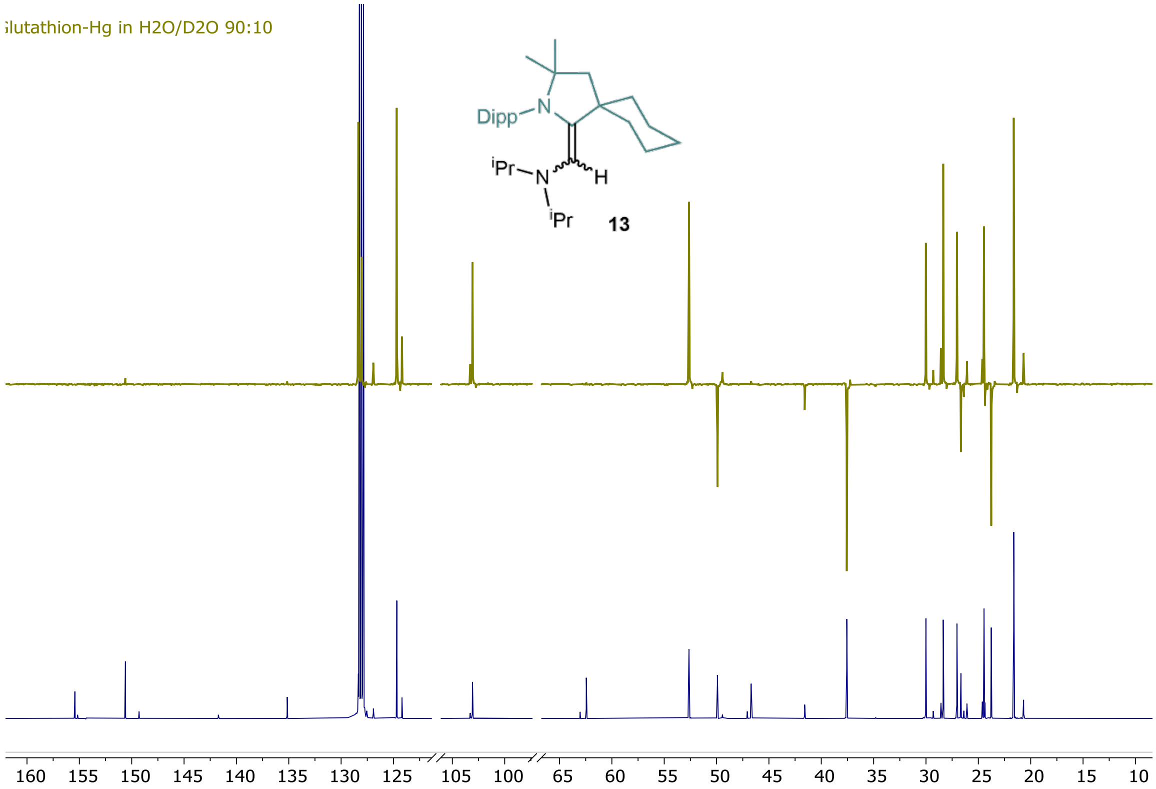

Figure S28. DEPT 135 (126 MHz) spectrum (yellow) and $^{13}C\{^1H\}$ NMR (126 MHz) spectrum (blue) of **13** in C_6D_6.

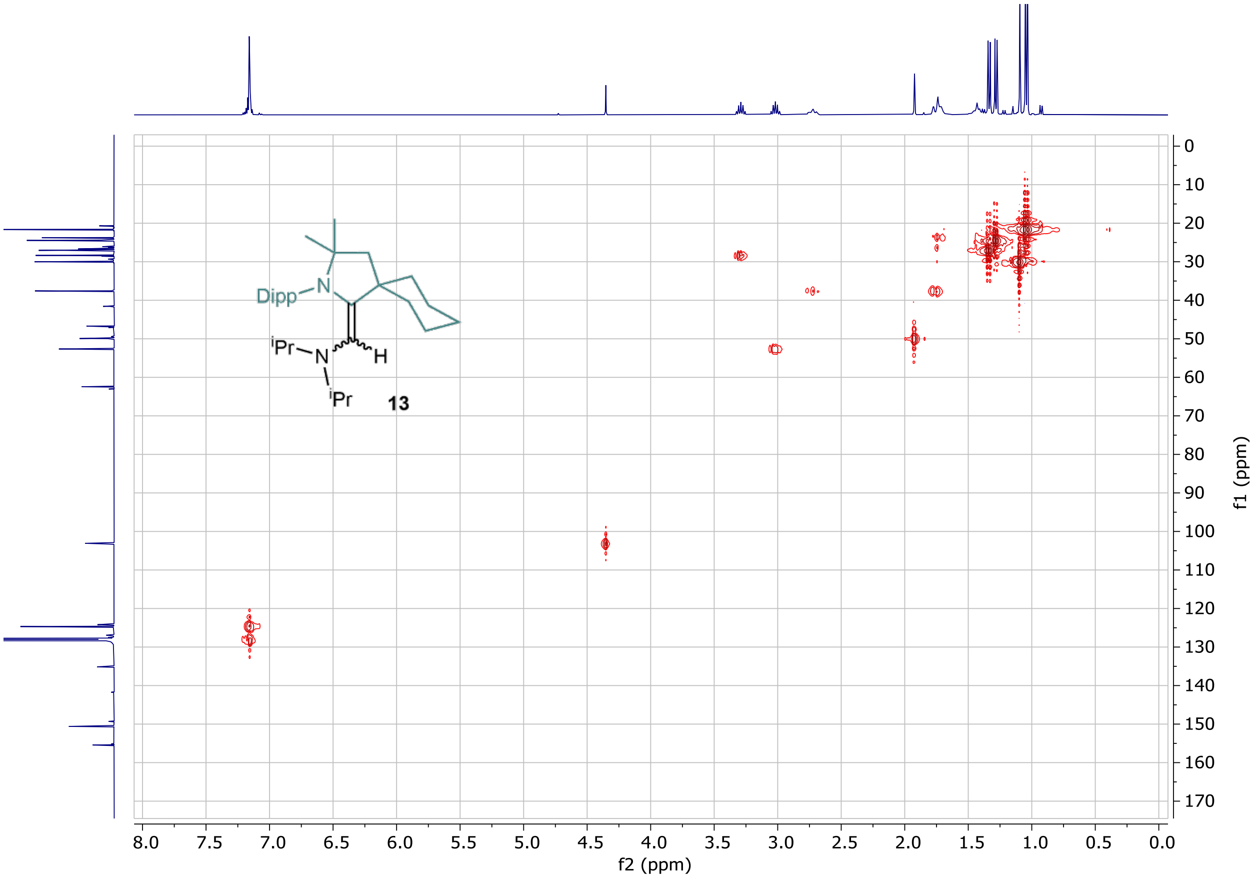

Figure S29. HMQC (400 MHz) spectrum of **13** in C_6D_6.

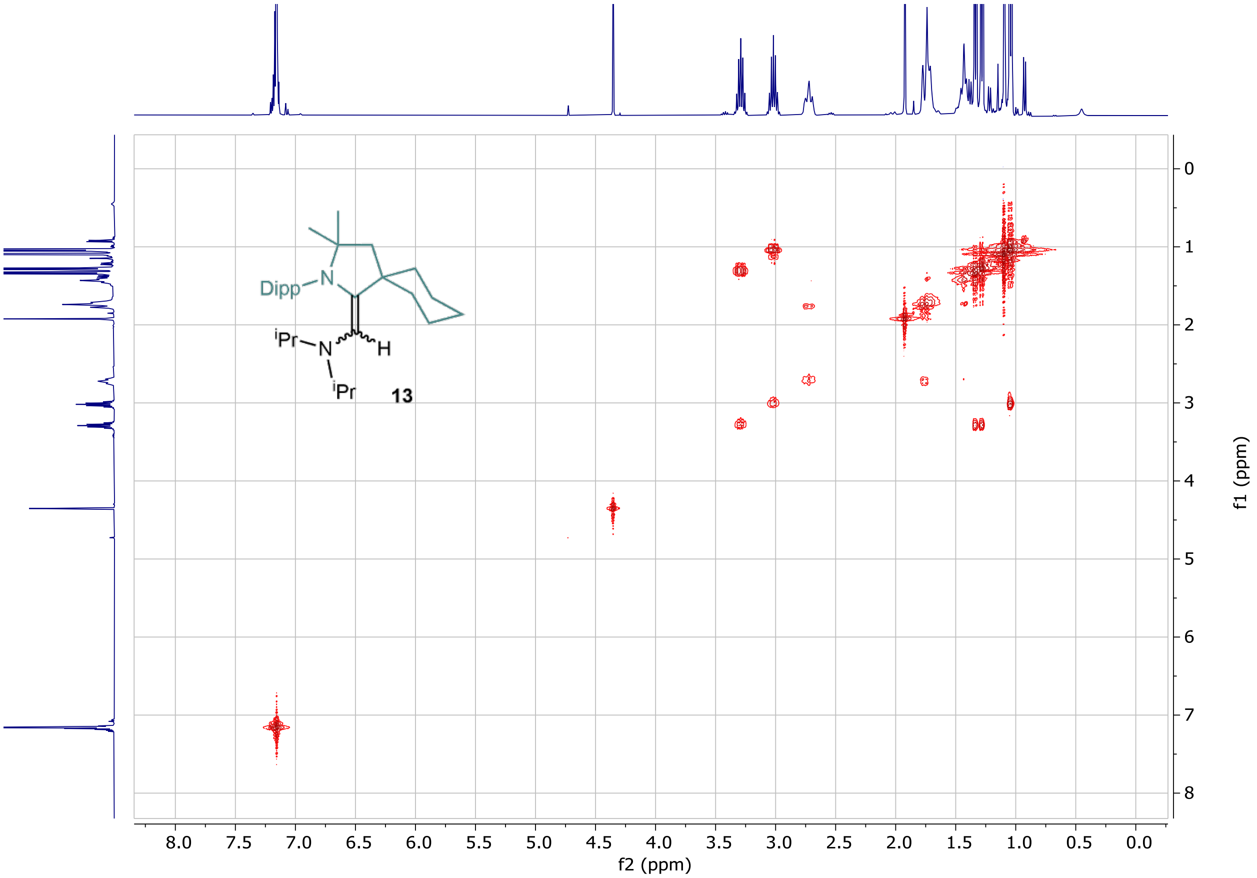

Figure S30. COSY (400 MHz) spectrum of **13** in C_6D_6.

IV. UV-Visible analysis

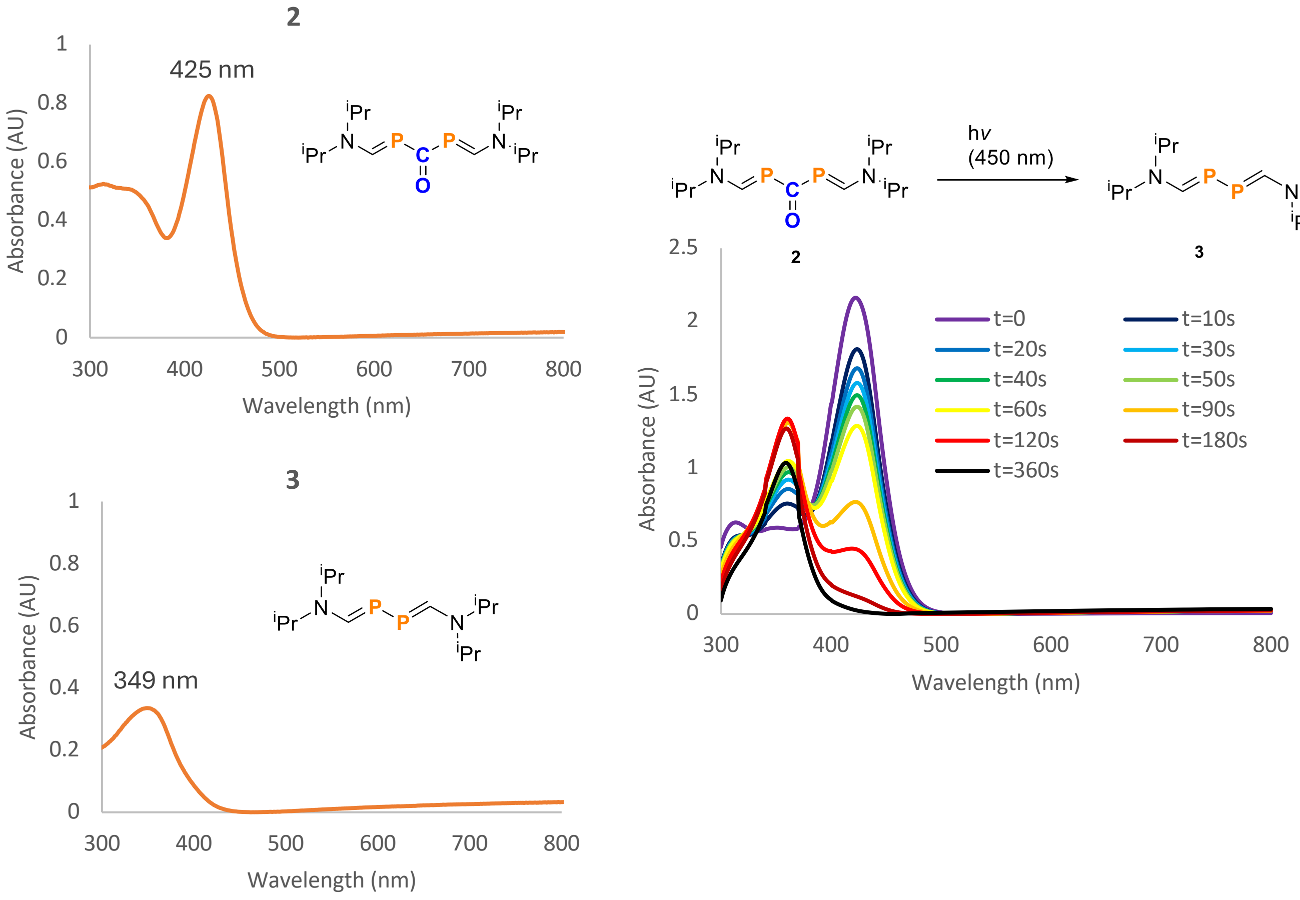

Figure S31. UV-Vis spectra of **2** (0.1 mM in THF) (top left), **3** (0.1 mM in THF) (bottom left), and spectra at different moments of the photo-conversion of **2** into **3** (right).

To help rationalize the photo-conversion of **2** into **3**, we monitored the absorbance of **3** during the irradiation at its maximum absorbance.

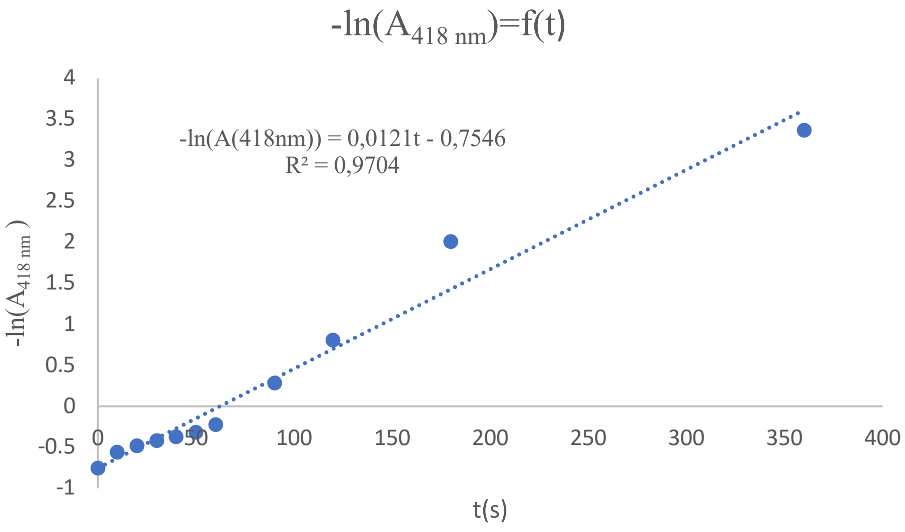

Hypothesis of a first-order kinetic:

$$-\frac{\mathrm{d}[\mathbf{2}]}{\mathrm{dt}} = k[\mathbf{2}]$$

$$Beer - Lambert\ law\text{: } A_{418\ nm} = \varepsilon(\mathbf{2})l[\mathbf{2}]$$

$$\rightarrow -\frac{\mathrm{d}A_{418\ nm}}{\mathrm{dt}} = kA_{418\ nm}$$

$$\rightarrow -\ln(A_{418\ nm}) = f(t) = kt + Constant$$

To confront our hypothesis, we realized the linear regression -ln($A_{418\ \mathrm{nm}}$) = f(t) and obtained a constant $k(T = 298K) = 0.0121\ s^{-1}$ with an excellent correlation coefficient of R^2=0.9704 which is in line with our working hypothesis.

V. X-ray crystallographic data

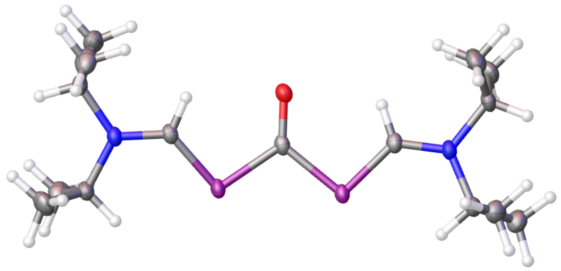

Table S32. Crystal data and structure refinement for **2** (CCDC 2367862)

Identification code	p(co)p	
Empirical formula	C15 H30 N2 O P2	
Formula weight	316.35	
Temperature	100.15 K	
Wavelength	1.54178 Å	
Crystal system	Monoclinic	
Space group	P 1 21/c 1	
Unit cell dimensions	a = 12.7343(6) Å	α= 90°.
	b = 11.6579(5) Å	β= 113.366(2)°.
	c = 14.1462(6) Å	γ = 90°.
Volume	1927.85(15) Å^3	
Z	4	
Density (calculated)	1.090 Mg/m^3	
Absorption coefficient	2.029 mm^{-1}	
F(000)	688	
Crystal size	0.2 x 0.1 x 0.1 mm^3	
Theta range for data collection	3.781 to 68.251°.	
Index ranges	-14<=h<=15, -14<=k<=14, -17<=l<=17	
Reflections collected	38129	
Independent reflections	3469 [R(int) = 0.0600]	
Completeness to theta = 67.679°	98.6 %	
Absorption correction	Semi-empirical from equivalents	
Max. and min. transmission	0.6617 and 0.5063	
Refinement method	Full-matrix least-squares on F^2	
Data / restraints / parameters	3469 / 0 / 195	
Goodness-of-fit on F^2	1.049	

Final R indices [I>2sigma(I)]	R1 = 0.0296, wR2 = 0.0753
R indices (all data)	R1 = 0.0333, wR2 = 0.0778
Extinction coefficient	n/a
Largest diff. peak and hole	0.359 and -0.151 e.Å^{-3}

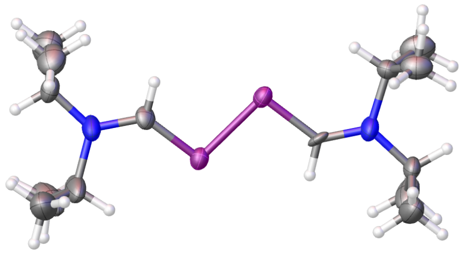

Table S33. Crystal data and structure refinement for **3** (CCDC 2367863).

Identification code	bis(carbene)p2	
Empirical formula	C14 H30 N2 P2	
Formula weight	288.34	
Temperature	100 K	
Wavelength	0.71073 Å	
Crystal system	Monoclinic	
Space group	P 1 21/n 1	
Unit cell dimensions	a = 11.7119(16) Å	α= 90°.
	b = 11.7054(15) Å	β= 113.731(4)°.
	c = 14.533(2) Å	γ = 90°.
Volume	1824.0(4) Å^3	
Z	4	
Density (calculated)	1.050 Mg/m^3	
Absorption coefficient	0.228 mm^{-1}	
F(000)	632	
Crystal size	0.15 x 0.12 x 0.08 mm^3	
Theta range for data collection	2.576 to 25.560°.	
Index ranges	-14<=h<=14, -14<=k<=14, -17<=l<=17	
Reflections collected	79435	
Independent reflections	3363 [R(int) = 0.0771]	
Completeness to theta = 25.242°	99.9 %	
Absorption correction	Semi-empirical from equivalents	
Max. and min. transmission	0.5547 and 0.5293	
Refinement method	Full-matrix least-squares on F^2	
Data / restraints / parameters	3363 / 261 / 348	
Goodness-of-fit on F^2	1.060	
Final R indices [I>2sigma(I)]	R1 = 0.0473, wR2 = 0.1110	
R indices (all data)	R1 = 0.0641, wR2 = 0.1220	
Extinction coefficient	n/a	

Largest diff. peak and hole	0.444 and -0.218 e.Å^{-3}

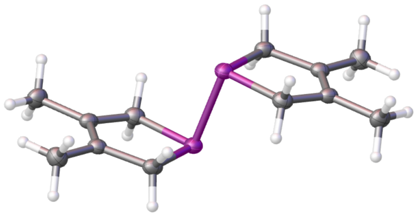

Table S34. Crystal data and structure refinement for **7** (CCDC 2367864).

Identification code	bis(phospholine)	
Empirical formula	C12 H20 P2	
Formula weight	226.22	
Temperature	100.00 K	
Wavelength	0.71073 Å	
Crystal system	Triclinic	
Space group	P-1	
Unit cell dimensions	a = 5.1758(3) Å	α= 88.010(2)°.
	b = 5.3182(3) Å	β= 83.733(2)°.
	c = 11.4365(6) Å	γ = 80.367(2)°.
Volume	308.46(3) $Å^3$	
Z	1	
Density (calculated)	1.218 Mg/m^3	
Absorption coefficient	0.315 mm^{-1}	
F(000)	122	
Crystal size	0.15 x 0.15 x 0.12 mm^3	
Theta range for data collection	3.585 to 25.636°.	
Index ranges	-6<=h<=6, -6<=k<=6, -13<=l<=13	
Reflections collected	2333	
Independent reflections	1167 [R(int) = 0.0482]	
Completeness to theta = 25.242°	99.8 %	
Absorption correction	None	
Refinement method	Full-matrix least-squares on F^2	
Data / restraints / parameters	1167 / 0 / 66	
Goodness-of-fit on F^2	1.142	
Final R indices [I>2sigma(I)]	R1 = 0.0490, wR2 = 0.1255	
R indices (all data)	R1 = 0.0528, wR2 = 0.1272	
Extinction coefficient	n/a	
Largest diff. peak and hole	0.443 and -0.413 e.$Å^{-3}$	

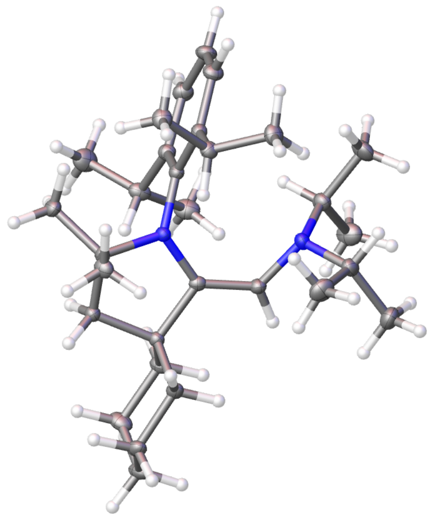

Table S35. Crystal data and structure refinement for **13** (CCDC 2367865).

Identification code	dimer_aminocarbene_caac	
Empirical formula	C30 H50 N2	
Formula weight	438.72	
Temperature	100.15 K	
Wavelength	1.54178 Å	
Crystal system	Monoclinic	
Space group	P 1 21/n 1	
Unit cell dimensions	a = 10.5119(4) Å	α= 90°.
	b = 15.7592(6) Å	β= 100.3031(10)°.
	c = 16.7454(6) Å	γ = 90°.
Volume	2729.30(18) $Å^3$	
Z	4	
Density (calculated)	1.068 Mg/m^3	
Absorption coefficient	0.451 mm^{-1}	
F(000)	976	
Crystal size	0.14 x 0.095 x 0.08 mm^3	
Theta range for data collection	3.881 to 68.245°.	
Index ranges	-12<=h<=12, -18<=k<=18, -17<=l<=20	
Reflections collected	34037	
Independent reflections	4985 [R(int) = 0.0394]	
Completeness to theta = 67.679°	99.9 %	
Absorption correction	Semi-empirical from equivalents	
Max. and min. transmission	0.6617 and 0.5979	

Refinement method	Full-matrix least-squares on F^2
Data / restraints / parameters	4985 / 0 / 299
Goodness-of-fit on F^2	1.024
Final R indices [I>2sigma(I)]	R1 = 0.0380, wR2 = 0.0971
R indices (all data)	R1 = 0.0396, wR2 = 0.0988
Extinction coefficient	n/a
Largest diff. peak and hole	0.310 and -0.204 e.Å^{-3}

VI. References

[i] Jazzar, R.; Dewhurst, R. D.; Bourg, J.-B.; Donnadieu, B.; Canac, Y.; Bertrand, G. Intramolecular "Hydroiminiumation" of Alkenes: Application to the Synthesis of Conjugate Acids of Cyclic Alkyl Amino Carbenes (CAACs). *Angew. Chem. Int. Ed.* **2007**, *46*(16), 2899-2902.

[ii] Quin, L. D.; Szewczyk, J. The Formation of P(III) Products From Phosphinamides with Silicon Hydrides. *Phosphorus and Sulfur*. **1984**, *21*, 161-170.
